# Hippocampus-Based Mitochondrial Respiratory Function Decline Is Responsible for Perioperative Neurocognitive Disorders

**DOI:** 10.3389/fnagi.2022.772066

**Published:** 2022-02-09

**Authors:** Keqiang He, Juan Zhang, Wei Zhang, Sheng Wang, Dingfeng Li, Xiaolin Ma, Xiaofan Wu, Xiaoqing Chai, Qiang Liu

**Affiliations:** ^1^Department of Anesthesiology, The First Affiliated Hospital of USTC, Division of Life Sciences and Medicine, University of Science and Technology of China, Hefei, China; ^2^Institute on Aging and Brain Disorders, The First Affiliated Hospital of USTC, Division of Life Sciences and Medicine, University of Science and Technology of China, Hefei, China; ^3^Biomedical Sciences and Health Laboratory of Anhui Province, University of Science and Technology of China, Hefei, China; ^4^National Synchrotron Radiation Laboratory, University of Science and Technology of China, Hefei, China; ^5^CAS Center for Excellence in Animal Evolution and Genetics, Chinese Academy of Sciences, Kunming, China

**Keywords:** perioperative neurocognitive disorders, postoperative cognitive dysfunction, neuroinflammation, mitochondrial respiratory chain complex, respiratory function

## Abstract

Perioperative neurocognitive disorders (PNDs) are a type of cognitive dysfunction occurring with a higher incidence in elderly patients. However, the pathological mechanism of PND and effective treatment remain elusive. We generated a PND mouse model by providing wild-type mice with surgical trauma; in our case, we used tibial fracture to investigate PND pathology. Mice aged 7–8 months were randomly divided into two groups: the surgery (tibial fracture) group and the control (sham) group. All mice were subjected to anesthesia. We examined the transcriptome-wide response in the hippocampus, a brain region that is tightly associated with memory formation, of control mice and mice subjected to surgical trauma at day 1 and day 3 after the surgical procedure. We observed reduced transcript levels of respiratory complex components as early as day 1 after surgery, and subsequent protein changes were found at day 3 after surgical trauma. Consequently, the activities of respiratory complexes were reduced, and adenosine triphosphate (ATP) production was decreased in the hippocampus of mice with surgical operations, supporting that respiratory chain function was impaired. In support of these conclusions, the mitochondrial membrane potential (MMP) levels were decreased, and the reactive oxygen species (ROS) levels were significantly increased. Mechanistically, we demonstrated that surgery induced a significant increase in cytokine IL-1β levels at day 1 after surgery, which concomitantly occurred with transcript changes in respiratory complex components. We further uncovered that transcription factors PGC-1α and NRF-1 were responsible for the observed transcript changes in mitochondrial complex components. Importantly, HT22 cells treated with the cytokine IL-1β resulted in similar reductions in PGC-1α and NRF-1, leading to a reduction of both the transcript and protein levels of respiratory complex subunits. Consequently, respiratory function was impaired in HT22 cells treated with IL-1β. Taken together, we demonstrated that reductions in respiratory complex components and subsequent impairment in mitochondrial functions serve as a novel mechanism for PND pathology, providing a potential therapeutic target for PND treatment.

## Introduction

Perioperative neurocognitive disorders (PNDs), including postoperative delirium (POD) and postoperative cognitive dysfunction (POCD) (Evered et al., 2018), are serious complications that affect up to 50% of surgical patients, especially those who are over 65 years old, for whom there is a lack of effective therapeutic treatments. It has been reported that the incidence of PND varies from 41–75% at 7 days to 18–45% at 3 months post-surgery (Zuo et al., 2020). Many major surgeries, such as hip or knee replacement, spinal operation, lower extremity arterial bypass operation, and open or laparoscopic colectomy, could lead to PND (Daiello et al., 2019). In fact, how anesthesia or surgery induces PND remains elusive (Eckenhoff et al., 2020). Currently, many reports speculate that neuroinflammation could be responsible for PND (Cibelli et al., 2010; Terrando et al., 2010; Degos et al., 2013; Hu et al., 2018; Ni et al., 2018; Skvarc et al., 2018; Saxena et al., 2019; Subramaniyan and Terrando, 2019; Yang et al., 2019, 2020). Patients who develop PND display increased proinflammatory cytokines in their cerebrospinal fluid (CSF) after anesthesia and surgery, suggesting a role of neuroinflammation in the pathology of PND (Hirsch et al., 2016). However, treatment with antibiotics prevents only a portion of model animals from PND induced by major surgeries (Liang et al., 2018). Additionally, corticosteroids, an anti-inflammatory medicine, could induce PND in a portion of patients over 65 years of age (Berger et al., 2018). These findings suggest that a mechanism other than neuroinflammation must exist.

Neuronal activity in the brain depends on the high energetic support provided by adenosine triphosphate (ATP), which is predominantly produced by mitochondria. Mitochondria-based energy metabolism is essential for cognitive function (Hebert-Chatelain et al., 2016). Neurons are highly specialized postmitotic cells that are inherently dependent on mitochondria to accommodate their high bioenergetic demand. Emerging evidence demonstrates that mitochondria are crucial for adult neurogenesis, which in turn contributes to cognitive processes in the mature brain (Khacho et al., 2019). Mitochondrial dysfunction is also tightly associated with various cognitive impairment-related disorders, such as Alzheimer’s disease (Swerdlow, 2018), Parkinson’s disease (Macdonald et al., 2018), and diabetes-associated cognitive impairment (Ma et al., 2020).

In this study, we adopted a classical mouse model of PND by conducting a surgical procedure of tibial fracture (Terrando et al., 2011; Hu et al., 2018; Xiong et al., 2018). The cognition of PND mice was assessed by conducting a series of behavioral tasks. We demonstrated that both the transcript and protein levels of mitochondrial respiratory complexes are significantly reduced in the hippocampus of PND model mice. These changes in respiratory complexes lead to decreased respiratory function in the hippocampus of PND model mice. Therefore, we defined a novel mechanism of PND pathology, where cognitive function decline results from the downregulation of respiratory chain function. This study also provides a potential therapeutic intervention for PND.

## Materials and Methods

### Animals

Male C57BL/6 wild-type mice aged 7–8 months were purchased from Vital River Laboratory Animal Technology [Beijing, China; Permit Number: SCXK (ZHE) 2019-0001]. All experimental protocols were approved by the Animal Studies Committee at the University of Science and Technology, Hefei, China. The animals were given *ad libitum* access to food and water. A 12:12-h light:dark cycle (09:00 a.m. to 09:00 p.m.) was followed in an air-conditioned environment. Animals were randomly divided into groups prior to any procedure or treatment.

### Establishment of Perioperative Neurocognitive Disorder Mouse Model

Mice were randomly assigned to two groups: the control (sham) and surgery (tibial fracture) groups. Control mice were subjected to anesthesia only, and the surgery group was subjected to anesthesia followed by tibial fracture operation. Mice were anesthetized with 2.1% isoflurane (RWD Life Science) using a commercially available rodent inhalation anesthesia apparatus (MIDTRX VIP2000, Midmark, Dayton, OH, United States) (Terrando et al., 2011; Hu et al., 2018). Tramadol (30 mg/kg) was subcutaneously administered to mice for analgesia immediately after anesthetic induction and prior to surgery (Xiang et al., 2019). Mice were subjected to an aseptic open tibial shaft fracture, and the fracture was managed by intramedullary nailing. During the surgery, the body temperature was maintained at 37°C using a heating lamp and a warming pad. Control mice received the same dose of anesthesia and analgesia but without tibial fracture procedures. The full procedure from the administration of anesthesia to the end of surgery lasted no longer than 15 min.

### RNA Isolation, Reverse Transcription, and Quantitative Polymerase Chain Reaction (PCR)

Hippocampal tissues were isolated from the brains of both control and surgery mice on day 1 post-surgery. Total RNA was extracted from hippocampal tissues using TRIzol (Invitrogen) and subjected to DNase I digestion for genomic DNA removal (Raihan et al., 2019). Reverse transcription was conducted using HiScript II Reverse Transcriptase (Vazyme) following the instructions of the manufacturer. Quantitative polymerase chain reaction (qPCR) was performed with AceQ qPCR SYBR Green Master Mix (Vazyme) on a Light Cycler 96 (Roche) instrument according to standard procedures. The real-time value for each sample was averaged and compared using the CT method, where the amount of target RNA (2^–ΔΔCT^) was normalized to a reference (ΔCT). The relative levels in the surgery group over the control group were plotted accordingly. qPCR detection primers are listed in Table 1.

**TABLE 1:** The forward and reverse primers for Quantitative PCR (qPCR) detection.

Gene	Primers	Sequence (5′ to 3′)
Cox5a	Forward primer	GCCGCTGTCTGTTCCATTC
Cox5a	**Reverse primer**	GCATCAATGTCTGGCTTGTTGAA
Ndufs6	Forward primer	GGGGAAAAGATCACGCATACC
Ndufs6	**Reverse primer**	CAAAACGAACCCTCCTGTAGTC
Ndufb8	Forward primer	TGTTGCCGGGGTCATATCCTA
Ndufb8	**Reverse primer**	AGCATCGGGTAGTCGCCATA
Ndufa1	Forward primer	ATGTGGTTCGAGATTCTCCCT
Ndufa1	**Reverse primer**	TGGTACTGAACACGAGCAACT
Cox4i1	Forward primer	ATTGGCAAGAGAGCCATTTCTAC
Cox4i1	**Reverse primer**	CACGCCGATCAGCGTAAGT
Ndufa2	Forward primer	TTGCGTGAGATTCGCGTTCA
Ndufa2	**Reverse primer**	ATTCGCGGATCAGAATGGGC
Ndufs7	Forward primer	GTTCATCAGAGTGTAGCCACTG
Ndufs7	**Reverse primer**	CAGGCCGAAGGTCATAGGC
Atp5d	Forward primer	TGCTTCAGGCGCGTACATAC
Atp5d	**Reverse primer**	CACTTGCTTGACGTTGGCA
Uqcr10	Forward primer	ATCCCTTCGCGCCTGTACT
Uqcr10	**Reverse primer**	GTGCTCGTAGATCGCGTCT
Ndufa6	Forward primer	GGTGAAACAAGGACGGGATA
Ndufa6	**Reverse primer**	GGAAAAACCGCATAACGTGT
β-actin	Forward primer	GGCTGTATTCCCCTCCATCG
β-actin	**Reverse primer**	CCAGTTGGTAACAATGCCATGT
Cox11	Forward primer	AATGCTGACGTTCATGCCAG
Cox11	**Reverse primer**	ACTGTCCAGCTTCAAACGGT
Atp5e	Forward primer	CAGGCTGGACTCAGCTACATC
Atp5e	**Reverse primer**	CCGAAGTCTTCTCAGCGTTC
Atp5k	Forward primer	CGGTTCAGGTCTCTCCACTC
Atp5k	**Reverse primer**	CCGCCAGTTCTCTCTCAATC
Atp6v1f	Forward primer	GACACGGTGACTGGTTTCCT
Atp6v1f	**Reverse primer**	CGAACCATCTCTGCGATGTA
Ndufb10	Forward primer	TGCCAAGAACCGAACCTACT
Ndufb10	**Reverse primer**	TGGCACAGTTCTGCTGGTAG
Cox8a	Forward primer	ATGTCTGTCCTGACGCCACT
Cox8a	**Reverse primer**	CAGGCAGAAGACAACACACG
Cox7c	Forward primer	GAGTATCCGGAGGTTCACGA
Cox7c	**Reverse primer**	TAAAGAAAGGTGCGGCAAAC
Cox6a1	Forward primer	AAGGCCCTCACCTACTTCGT
Cox6a1	**Reverse primer**	TTCACATGAGGGTTGTGGAA
Uqcr11	Forward primer	TGCTGAGCAGGTTTCTAGGC
Uqcr11	**Reverse primer**	CCTTCTTAAACTTGCCGTTGA
Ndufb9	Forward primer	GCCCGGTTTGAAGAACATAA
Ndufb9	**Reverse primer**	GCACCATTCTGGAACCTTGT

### Next-Generation Sequencing

Hippocampal tissues were isolated from the brains of both control and surgery mice on day 1 post-surgery. Total RNA was extracted from hippocampal tissues (2 mice in each group). Total mRNA was enriched by oligo (dT) beads, followed by fragmentation and reverse transcription with random primers. The remaining mRNA was removed by RNase H treatment. cDNA was purified, and the adapters were ligated at the 5′ and 3′ ends. Ligated cDNA was PCR amplified and subjected to an Illumina NovaSeq System for 150 nt paired-end sequencing (Annoroad Gene Technology). The raw RNA-Seq data were filtered to obtain clean reads, followed by mapping to the mouse reference genome (mm10) using HISAT.

### Kyoto Encyclopedia of Genes and Genomes and Gene Ontology Analysis

Kyoto Encyclopedia of Genes and Genomes (KEGG) pathway enrichment analyses and Gene Ontology (GO) functional annotation were performed for differentially expressed genes in surgery over control groups using Database for Annotation, Visualization, and Integrated Discovery (DAVID v6.8).

### Purification of Mitochondria From Hippocampal Tissues

Mitochondria were purified from hippocampal tissues using a commercial kit (Beyotime, Cat#:C3606). Briefly, fresh tissues were homogenized in ice-chilled isolation buffer (1:10, w/v) using Dounce homogenizers and centrifuged at 1,000*g* for 5 min at 4°C. Supernatants were saved and transferred to an EP tube, followed by centrifugation at 3,500*g* for 10 min at 4°C. The resulting sediment was considered mitochondria. The protein concentration in mitochondrial fractions was determined using a BCA protein assay kit (Thermo Scientific, Cat #: P0010S).

### Enzyme-Linked Immunosorbent Assay (ELISA)

Mice were perfused with phosphate-buffered saline (PBS), and the hippocampal tissues were harvested. Proteins were extracted with RIPA buffer (Beyotime, Cat#: P0013B) containing 1% PMSF (Beyotime, Cat#: ST506) and 1% cocktail protease inhibitor (MCE, Cat#: C0001), followed by protein concentration determination with a BCA protein assay kit (Thermo Scientific, Cat #: P0010S). Levels of IL-6 (R&D Systems, Cat#: EMC004) and IL-1β (NeoBioscience, Cat#: EMC001b) were measured in hippocampal tissues by an Enzyme-linked immunosorbent assay (ELISA)-based approach according to the protocols of the manufacturer.

### Plasmid Construction

The coding sequences of the PGC-1α gene were synthesized and then inserted into the pcDNA 3.1 vector (General Biol).

### Immunoblotting and Densiometric Analysis

Proteins were extracted from hippocampal tissues or cells as described in the “ELISA” section. Equal amounts of proteins were resolved by SDS-PAGE electrophoresis and then transferred from SDS-PAGE gels to nitrocellulose membranes (Poll, Cat# 66485). The membranes were incubated with 5% non-fat milk at room temperature for 1 h, followed by incubation with primary antibodies at 4°C overnight. The following antibodies were used: polyclonal rabbit anti-IL-1β (1:1,000, Proteintech, Cat#:16806-1-AP), monoclonal mouse anti-PGC-1α (1:5,000, Proteintech, Cat#:66369-1-Ig), polyclonal rabbit anti-NRF-1 (1:1,000, Proteintech, Cat#:12482-1-AP), polyclonal rabbit anti-Cox5a (1:500, Proteintech), polyclonal rabbit anti-ATP5d (1:1000, Proteintech, Cat#:14893-1-AP), polyclonal rabbit anti-ATP5k (1:500, Proteintech, Cat#:16483-1-AP), polyclonal rabbit anti-Ndufs6 (1:500, Proteintech, Cat#:14417-1-AP), monoclonal mouse anti-Ndufb8 (1:10,000, Proteintech, Cat#:67690-1-Ig), polyclonal rabbit anti-Ndufb10 (1:1,000, Proteintech, Cat#:15589-1-AP), and monoclonal mouse β-actin antibody (1:5,000, Affinity, Cat#:AF7018). The immunoreactive bands were visualized by enhanced chemiluminescence (Thermo Scientific) and detected by Chemiscope (CLiNX). Immunoreactive bands were quantified using ImageJ software (NIH).

### Mitochondrial Respiratory Complex Activity Determination

Activities of nicotinamide adenine dinucleotide (NADH) dehydrogenase (complex I) and cytochrome C oxidase (complex IV) were measured by commercially available kits (Solarbio, Cat#: BC0515 and Cat#: BC0945) according to the instructions of the manufacturer. Briefly, reaction buffer was added to purified mitochondria, and this reaction mixture was transferred to a 96-well plate, followed by reading on a SpectraMax i3x microplate reader (Molecular Devices). The absorbance of the reaction mixture was measured at 340 nm for complex I or 550 nm for complex IV.

### Determination of Adenosine Triphosphate Levels

Adenosine triphosphate levels were measured using a firefly luciferase-based ATP assay kit (Beyotime, Cat#: S0026) according to the instructions of the manufacturer. Briefly, hippocampal tissues were lysed in ice-cold lysis buffer, homogenized with a Dounce homogenizer on ice, and centrifuged at 12,000*g* for 5 min at 4°C. Twenty microliters of samples or standards were added to each well containing 100 μl of working solutions. Luciferase levels were measured using a SpectraMax i3x microplate reader (Molecular Devices).

### Determination of Mitochondrial Membrane Potential

Mitochondrial membrane potential (MMP) levels were measured using a commercially available kit (Beyotime, C2006). Briefly, a 5,5′,6,6′-tetrachloro-1,1′,3,3′ tetraethylbenzimidazolyl-carbocyanine iodide (JC-1) probe was incubated with purified mitochondria prepared from hippocampal tissues. Fluorescence for each sample was measured using SpectraMax i3x (Molecular Devices) at an excitation/emission wavelength of 485/590 nm.

### Determination of Reactive Oxygen Species

Reactive oxygen species (ROS) levels were measured using a commercially available kit (GENMED, Cat#: GMS10016.4). Briefly, cold GENMED diluent was quickly added to freshly dissected hippocampal tissues, followed by homogenization in a Dounce homogenizer on ice. The protein concentrations were determined using a BCA protein assay kit (Thermo Scientific, Cat #: P0010S). The working solution containing DCFHDA (100 μl) was added to each sample in a 96-well plate. Fluorescence was measured using a SpectraMax i3x microplate reader (Molecular Devices) at an excitation/emission wavelength of 490/520 nm.

Cells were incubated with an *in situ* DCFHDA probe at 37°C for 20 min according to the instructions of the manufacturer. ROS fluorescence signals were captured with a CCD camera mounted onto a U-CB5S microscope system (Olympus). Relative fluorescence intensities were calculated using ImageJ (NIH).

### Open Field Test

An open field task was conducted to evaluate anxiety behavior and general locomotor activity. Mice were subjected to the open field task on day 6 post-surgery. Each mouse was gently placed at the center of a black chamber (45 cm × 45 cm × 40 cm) for 5 min, and exploratory behavior was recorded using a video tracking system (Smart 3.0 software, Panlab, Spain). Locomotor activity was determined by the total distance traveled, and anxiety behavior was determined by the time spent in the center and edge of the chamber. The arena was cleaned with 75% alcohol before and after each test to avoid olfactory cues.

### Novel Object Recognition Test

The novel object recognition test was conducted as described previously, with minor modifications (Lueptow, 2017; Zhang et al., 2021). Before the test, mice were allowed to explore the test chamber (with a dimension of 45 cm × 45 cm × 40 cm; RWD Life Science) for 5 min/day for 2 days. This habituation session occurred at day 5 and day 6 after the operations. On day 7 after the operation, two identical objects (A + A) were presented to mice, followed by exploration for 10 min (training session). Four hours after the training session, mice were presented with two objects, one familiar object (A) and one novel object (B), in the same position for 10 min (test session). The exploring time for each object was recorded. Data were analyzed using Smart 3.0 software (Panlab, Spain). The cognition index was defined as the ratio of time spent exploring the novel object to the total time spent exploring both objects.

### Fear Conditioning Test

The fear conditioning test was used to assess contextual memory as described previously (Hu et al., 2018). Briefly, animals were trained to associate a conditional stimulus (tone) with an aversive, unconditional stimulus (foot shock). The behavioral test was conducted using a conditioning chamber (Soft maze Information Technology). Mice were placed in a test chamber and allowed to explore for 100 s and then presented with an auditory cue (75–80 dB, 5 kHz) for 20 s (conditional stimulus). A foot shock (0.75 mA) (unconditional stimulus) was administered overlapping with the conditional stimulus for 2 s. Auditory cues and foot shock were given to mice with the same paradigm after a 100 s break. The context test was conducted 24 h after the training session, during which no tones or foot shocks were provided to mice. Freezing behavior was recorded for both the training and test sessions, and freezing time was analyzed.

### Cell Culture, Transfection, and Treatment

HT22 cells were cultured in Dulbecco’s Modified Eagle Medium (DMEM) (HyClone), supplemented with 10% fetal bovine serum (FBS) (Biological Industries) and 1% penicillin–streptomycin (Beyotime) in a 5% CO_2_ incubator (Thermo Fisher Scientific) at 37°C. Plasmid transfection was performed when cells were approximately 70–80% confluent using Lipofectamine 2000 (Invitrogen). HT22 cells were treated with IL-1β for 48 h at a concentration of 10 ng/ml (MedChemExpress, HY-P7073). Cells were collected at the end of the treatment, and RNA or protein was extracted for further analysis. All experiments were repeated at least three times.

### Measurement of the Blood–Brain-Barrier Permeability

Evans blue (EB) (2%) in saline solution was injected through the caudal vein of mice (4 ml/kg). The amount of EB in the brain was measured according to the previously published work (Uyama et al., 1988). One hour after the injection, mice were perfused with PBS, and the brain tissue was homogenized in 50% trichloroacetic acid solution. Brain homogenates were then subjected to centrifugation at 10,000 rpm for 20 min at 4°C, and the supernatant was diluted in absolute ethanol. External standards of EB solution range from 1.95 to 125 ng/ml, and prepared samples were added to 96-well plates; fluorescence signals were detected with a CLARIOstar microplate reader (excitation: 620 nm, emission: 680 nm) (BMG LABTECH, Germany).

### Immunofluorescence Staining

Immunofluorescence staining was performed as described previously (Li et al., 2021). Cells were seeded on coverslips, washed 3 times with PBS, and fixed in 4% PFA for 15 min. Cells were permeabilized with PBS containing 0.2% Triton X-100 (PBST), followed by blocking with 2% BSA in PBST for 30 min. After blocking, cells were incubated with primary antibodies overnight at 4°C, followed by incubation with an Alexa-conjugated secondary antibody (Invitrogen). Fluorescence signals were captured with a CCD camera mounted onto a U-CB5S microscope system (Olympus). The following antibodies were used for immunofluorescence staining: monoclonal rabbit anti-Ndufs6 (1:200, Abcam, Cat#: ab195808), monoclonal mouse anti-PGC-1α (1:200, Proteintech, Cat#: 66369-1-Ig), and monoclonal rabbit anti-NRF-1 (1:200, Abcam, Cat#: ab175932).

### Statistical Analysis

All quantified data represent an average of at least triplicate samples. Statistical significance was determined by independent samples (unpaired) Student’s *t*-test (two-tailed) or one-way analysis of variance (ANOVA) in SPSS26.0 or GraphPad Prism 7.0. *P* < 0.05 was considered significant (indicated by an asterisk in the figures); *P* < 0.01 and *P* < 0.001 were indicated by two asterisks and three asterisks, respectively, in the figures.

## Results

### Perioperative Neurocognitive Disorder Mouse Model Establishment

Previous studies have shown that trauma-induced memory decline lasts longer in older mice than in younger mice (Eckenhoff et al., 2020); however, aged mice with an impaired blood–brain barrier (BBB) may also bring possible confounding factors from the circulation (Yang et al., 2017; Ni et al., 2018). Therefore, we selected middle-aged (aged 7–8 months) C57BL/6 male mice to conduct tibial fracture operations and to create a PND mouse model (Ackert-Bicknell et al., 2015; Wang et al., 2018). We first evaluated this PND mouse model by assessing their cognitive behaviors.

**FIGURE 1:**
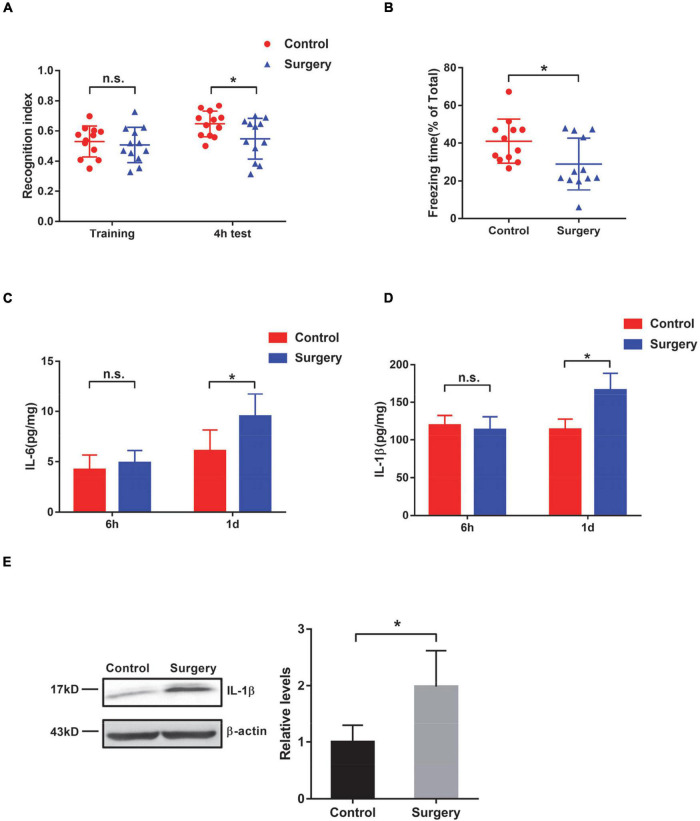
Evaluation of cognition and neuroinflammation in mice after tibial surgery. **(A)** Contextual memory was evaluated by the novel object recognition (NOR) task. The recognition index was calculated for control and surgery mice at the training phase and 4 h after the training (*n* = 12 per group). **(B)** Contextual memory was evaluated by the fear conditioning task. Freezing time was recorded, and the ratio of freezing time to the total testing time was calculated (*n* = 12 per group). **(C–E)** Wild-type (WT) mice were subjected to tibial surgery, and hippocampal tissues were collected at 6 h or 1 day post-surgery. Levels of IL-6 **(C)** and IL-1β **(D)** in the hippocampus were determined by an ELISA-based approach (*n* = 6–9 per group). Levels of IL-1β in the hippocampus were also determined by immunoblotting and densiometric analysis **(E)** (*n* = 4 per group). In this and subsequent figures, h represents hours and d represents days. *P < 0.05 by independent samples Student’s *t*-test; n.s. not significant; error bars denote the standard error of mean (SEM).

At 7 days after surgery, mice were subjected to novel object recognition (NOR) task for contextual memory assessment. The control and surgery mice showed no significant changes during the training session. Four hours after the training session, mice that underwent surgery exhibited significantly decreased time spent on exploring the novel object compared with the control mice that did not undergo surgery (Figure 1A), suggesting that contextual memory was impaired in the surgically treated mice. Consistently, the fear conditioning (FC) task also showed that the freezing time was significantly lower in surgery mice than in control mice, supporting fear-related contextual memory decline in PND mice (Figure 1B). Cognitive impairment of these mice with tibial fracture operation suggests that this PND model is successfully established. The open field test was carried out to evaluate the locomotor activity and anxiety behavior of mice. We found that control and PND mice exhibited similar locomotor activities and anxiety behavior (Supplementary Figures 1A,B).

A previous study demonstrated that surgery and anesthesia together induce significant reductions in BBB permeability in mice (Yang et al., 2017); therefore, we examined the permeability of the BBB in our PND model mice. We found that the surgery mice (surgery and anesthesia) showed higher BBB permeability than the control mice (anesthesia only), whereas the control mice exhibited similar BBB permeability as those mice that were subjected to neither anesthesia nor surgery treatment (naïve) (Supplementary Figure 1C). Interleukin-6 (IL-6) is a well-known cytokine that indicates the inflammatory status (Hu et al., 2018). In this study, we observed an increase in IL-6 in the hippocampus of PND mice at 1 day after tibial fracture, but not at an earlier time point (6 h after the operation) (Figure 1C). In addition to IL-6, we also detected a significant increase in IL-1β levels (Figures 1D,E), which is also considered an indicator of inflammation (Cibelli et al., 2010). These observed changes in IL-6 and IL-1β levels also support the successful establishment of this PND mouse model.

### Transcriptome Analysis of Hippocampal Tissues of Perioperative Neurocognitive Disorder Model Mice

**FIGURE 2:**
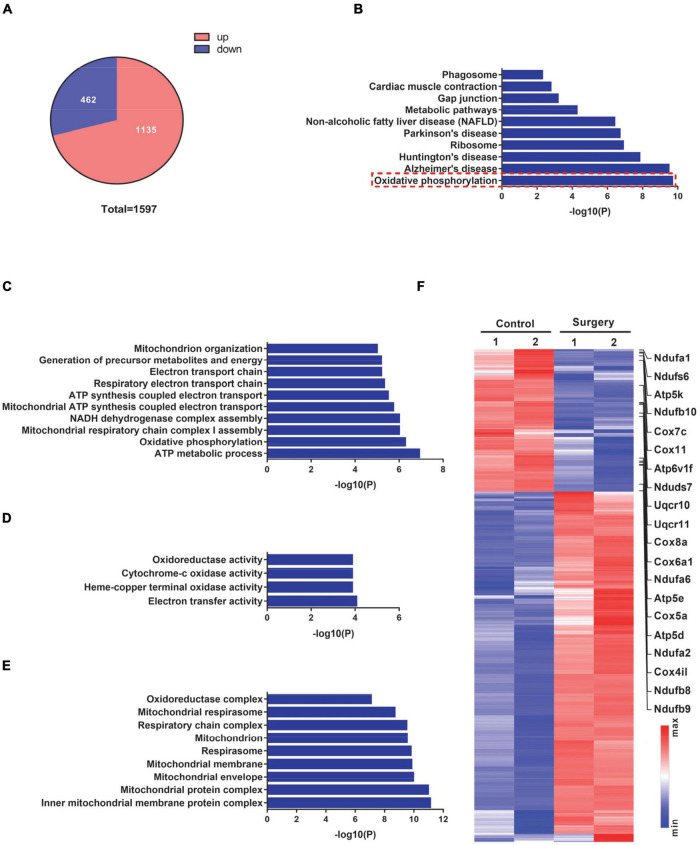
Transcriptome profiles of hippocampal tissues from control mice and mice at 1 day after surgery. **(A)** Pie chart demonstrating the total number of differentially expressed genes (DEGs) (fold-change cutoff = 1.5) and the number of significantly up- and downregulated genes (surgery vs. control). **(B)** Kyoto Encyclopedia of Genes and Genomes (KEGG) pathway analysis for significantly downregulated genes in **(A)**. **(C–E)** Biological process **(C)**, molecular function **(D)**, and cellular component **(E)** from Gene Ontology (GO) analysis for significantly downregulated genes in **(A)**. **(F)** DEGs in the surgery vs. control groups were visualized in a heatmap. Oxidative phosphorylation-related genes are indicated on the right side of this heatmap.

To investigate the impact of surgical trauma on postoperative cognition, we next conducted transcriptome analyses of the hippocampus isolated from control and PND mice. We detected a total of 1,597 differentially expressed genes (DEGs) in PND mice, among which 1,135 (71%) DEGs were significantly upregulated (fold change ≥ 1.5, PND vs. control) and 462 (29%) DEGs were significantly downregulated (fold change ≤ 0.67, PND vs. control) (Figure 2A).

We next conducted a KEGG pathway analysis to explore the potential pathway that is involved in the defective cognitive function of PND mice. These significantly downregulated DEGs in the KEGG pathway analysis exhibited enriched functional annotation related to oxidative phosphorylation and neurodegenerative diseases, such as Alzheimer’s disease, Parkinson’s disease, and Huntington’s disease (Figure 2B). Furthermore, we performed GO analyses on these significantly downregulated DEGs. The top terms from the biological process (BP) in the ontology were strongly related to mitochondrial functions, such as ATP metabolic process, oxidative phosphorylation, and mitochondrial respiratory chain complex assembly (Figure 2C). Similarly, terms from the molecular function (MF) in the ontology also showed a strong enrichment for mitochondria-related activities, such as oxidoreductase activity, cytochrome-c oxidase activity, and electron transfer activity (Figure 2D); and terms from the cellular component (CC) displayed a strong enrichment for mitochondrial structural components, such as inner mitochondrial membrane protein complex, mitochondrial protein complex, mitochondrial membrane, and respiratory chain complex (Figure 2E). A total of 1,597 DEGs were visualized in a heatmap, with mitochondrial respiratory complex-related genes labeled (Figure 2F). These findings suggest that operation-induced decreases in genes in PND mice are mostly related to mitochondrial respiratory functions.

### Perioperative Neurocognitive Disorder Mice Show Decreased Transcript Levels of Genes Encoding Respiratory Complex Subunits

**FIGURE 3:**
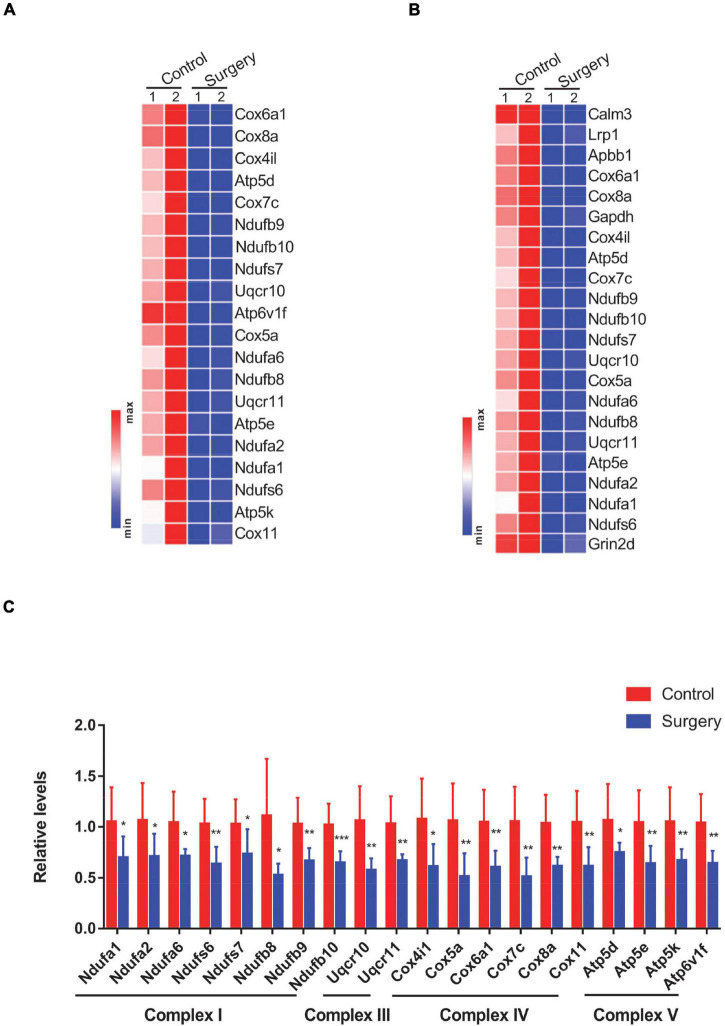
Differential expression of oxidative phosphorylation-related genes was visualized in heatmaps and validated by Quantitative PCR (qPCR). **(A,B)** Genes enriched in the “oxidative phosphorylation” **(A)** and “Alzheimer’s disease” **(B)** terms from Kyoto Encyclopedia of Genes and Genomes (KEGG) pathway analysis are visualized in heatmaps. **(C)** Levels of oxidative phosphorylation-related genes in respiratory chain complexes in control vs. surgery mice were determined by qPCR-based analysis. β-actin was included as an internal control. Data are presented as fold changes in the surgery over controls (*n* = 8 per group). *P < 0.05; **P < 0.01; ***P < 0.001 by independent samples Student’s *t*-test; error bars denote the SEM.

The differential expression pattern of significantly downregulated genes enriched in the top two terms from KEGG analysis was specifically visualized in heatmaps. Genes enriched in the “oxidative phosphorylation” term (*n* = 20) were mostly mitochondrial complex components (Figure 3A). Similarly, genes enriched in the “Alzheimer’s disease” term (*n* = 22) were predominantly mitochondrial complex components (Figure 3B), wherein 17 genes were found both in the “oxidative phosphorylation” term and in the “Alzheimer’s disease” term. This is not to our surprise, as mitochondrial dysfunction is extensively documented during Alzheimer’s disease progression (Swerdlow, 2018). Of note, 20 genes that were enriched in the “oxidative phosphorylation” term were related to NADH-dependent respiratory chain activity. Specifically, eight genes, namely, Ndufa1, Ndufa2, Ndufa6, Ndufs6, Ndufs7, Ndufb8, Ndufb9, and Ndufb10, encode subunits of complex I; two genes, namely, Uqcr10 and Uqcr11, encode subunits of complex III; six genes, namely, Cox4i1, Cox5a, Cox6a1, Cox7c, Cox8a, and Cox11, encode subunits of complex IV; and four genes, namely, Atp5d, Atp5e, Atp5k, and Atp6v1f, encode subunits of complex V. We next verified the expression of these 20 DEGs (Figure 3A) by a qPCR-based approach. Consistent with the RNA-Seq data, these genes were all significantly decreased in the hippocampus of PND mice, compared with the control mice (Figure 3C). Taken together, our findings suggest that PND mice show reduced mitochondrial complex levels in their hippocampus.

### Downregulation of the Transcription Factors Nuclear Respiratory Factor-1 and Peroxisome Proliferator-Activated Receptor-γ Coactivator-1α Is Responsible for the Reductions in Respiratory Complex Subunits

**FIGURE 4:**
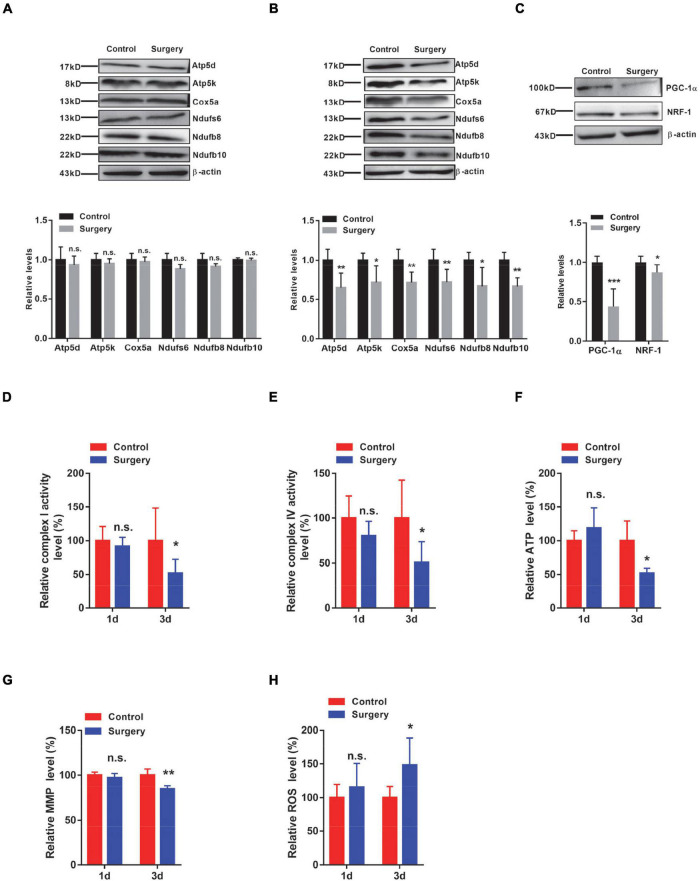
Respiratory complex levels and respiratory function are decreased in the Perioperative neurocognitive disorder (PND) mouse brain by downregulating both peroxisome proliferator-activated receptor-γ coactivator-1α (PGC-1α) and Nuclear respiratory factor-1 (NRF-1) levels. **(A,B)** Protein levels of Atp5d and Atp5k (complex V); Cox5a (complex IV); and Ndufs6, Ndufb8, and Ndufb10 (complex I) were determined in hippocampal tissues collected from control and surgery mice (1 day post-surgery) (*n* = 4) **(A)** or from control and surgery mice (3 days post-surgery) (*n* = 5) **(B)**, by immunoblotting and densiometric analysis. **(C)** Levels of PGC-1α and NRF-1 in the hippocampal tissues of control and surgery mice (1 day post-surgery) were determined by immunoblotting and densiometric analysis (*n* = 6). β-actin was included as an input control. **(D,E)** Hippocampal tissues were isolated from control or surgery mice (1 day or 3 days post-surgery). Enzymatic activities of NADH dehydrogenase (complex I) **(D)** and cytochrome C oxidase (complex IV) **(E)** were assessed by an ELISA-based approach (*n* = 8 per group). **(F–H)** Hippocampal tissues were isolated from control or surgery mice (1 day or 3 days post-surgery) (*n* = 5 per group). **(F)** Adenosine triphosphate (ATP) levels were assessed by an ELISA-based analysis. **(G)** Mitochondrial membrane potential (MMP) levels were determined by the JC-1 fluorescence probe-based approach. **(H)** Relative reactive oxygen species (ROS) levels were determined by the DCFHDA probe-based approach. *P < 0.05; **P < 0.01; ***P < 0.001 by independent samples Student’s *t*-test; n.s. not significant; error bars denote the SEM.

Recalling that transcript changes in respiratory complex components occurred at 1 day after surgery, we next assessed the protein levels of selected respiratory chain complex components, such as Atp5d and Atp5k from complex V; Cox5a from complex IV; and Ndufs6, Ndufb8, and Ndufb10 from complex I. Intriguingly, we did not observe significant changes in these respiratory complexes in the hippocampus of mice at 1 day after surgery (Figure 4A). However, we indeed observed significantly decreased protein levels of Atp5d, Atp5k, Cox5a, Ndufs6, Ndufb8, and Ndufb10 in the hippocampal tissues of PND mice at 3 days after surgery, compared with the control mice (Figure 4B). Densiometric analyses also support these notions (Figures 4A,B). Recalling that the levels of IL-1β were significantly increased at 1 day post-surgery, transcript, but not protein, changes in respiratory complex components were observed in the hippocampus of PND mice. These findings suggest that surgery-induced changes in respiratory complex levels are associated with IL-1β levels.

Peroxisome proliferator-activated receptor-γ coactivator-1α (PGC-1α) is a transcription factor that is strongly involved in the mitochondrial biogenesis process (Wang et al., 2015). We next examined the levels of PGC-1α and found that its levels were significantly decreased in the hippocampus of PND mice, compared with the control mice (Figure 4C). This conclusion was also evident by densiometric analysis (Figure 4C). Nuclear respiratory factor-1 (NRF-1) is another transcription factor that has been shown to be a key regulator of oxidative phosphorylation-related genes (Bouchez and Devin, 2019). We, therefore, examined the levels of NRF-1 and found that its levels were significantly reduced in the hippocampal tissues of PND mice compared with the same brain region of control mice (Figure 4C). Importantly, these surgery-induced changes in PGC-1α and NRF-1 were observed at 1 day post-surgery, indicating that the transcription factors PGC-1α and NRF-1 may participate in the regulation of respiratory complex component levels.

### Respiratory Complex Activity and Respiratory Function Are Impaired in Perioperative Neurocognitive Disorder Mice

Given the aforementioned changes in respiratory complex levels, we next examined the function of respiratory complexes by measuring the activities of respiratory enzymes. We found a significant decrease in the activities of both complex I and complex IV in the hippocampal tissues of PND mice at 3 days post-surgery, but not at 1 day post-surgery (Figures 4D,E). We next assessed the levels of ATP, which not only represents the activity of complex V but also reflects the function of the respiratory chain. We consistently observed significantly lower ATP levels in the hippocampus of PND mice at 3 days after surgery than in the same brain region of the control mice, whereas the hippocampus of mice at 1 day after surgery showed no significant difference in ATP levels (Figure 4F). Furthermore, we also evaluated the MMP and found that MMP levels were significantly reduced in the hippocampus of mice at 3 days after surgery, but not in the hippocampus of mice at 1 day after surgery (Figure 4G). Notably, the levels of ROS were significantly increased in the hippocampus of mice at 3 days after surgery, whereas no significant changes were detected in the hippocampus of mice at 1 day after surgery (Figure 4H). These findings suggest that altered respiratory complex levels lead to mitochondrial dysfunction in the hippocampus of PND mice in a time-dependent manner.

### Interleukin-1β Induces Reductions in Respiratory Chain Complex Components by Downregulating Peroxisome Proliferator-Activated Receptor-γ Coactivator-1α and Nuclear Respiratory Factor-1 *in vitro*

**FIGURE 5:**
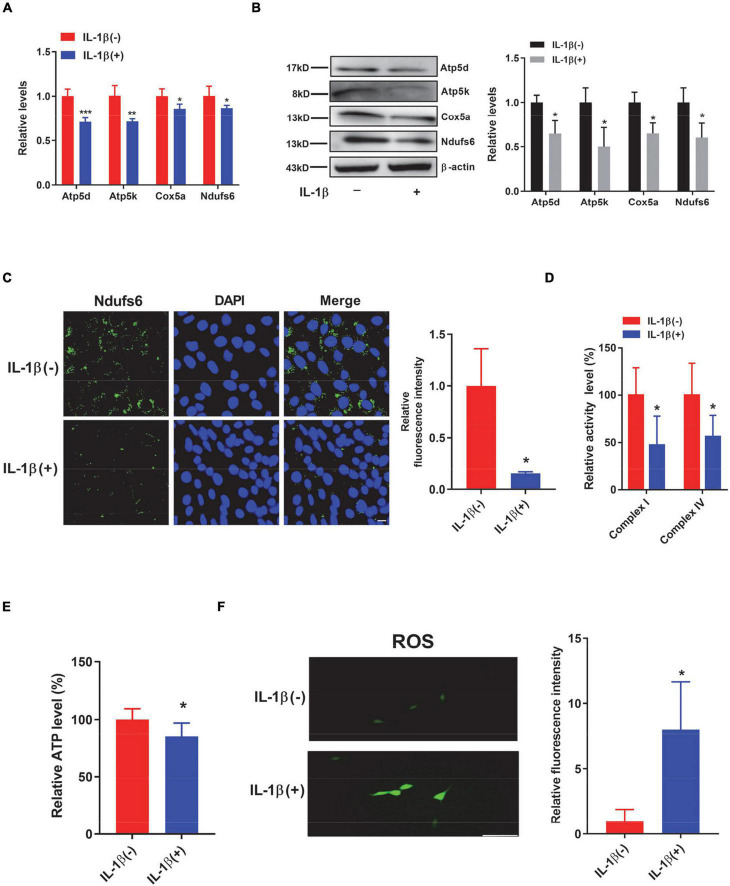
HT22 cells treated with interleukin-1β (IL-1β) show reduced respiratory complex levels and decreased mitochondrial respiratory function. **(A–F)** HT22 cells were treated with IL-1β for 48 h. Levels of selected respiratory chain components were assessed by Quantitative PCR (qPCR) (*n* = 8 per group) **(A)** and by immunoblotting and densiometric analysis **(B)** (*n* = 4 per group). **(C)** Representative immunofluorescence images of Ndufs6 (green) and DAPI (blue) in control and IL-1β-treated HT22 cells. Scale bar: 20 μm. Relative immunofluorescence intensity was plotted in IL-1β-treated HT22 cells over control HT22 cells (*n* = 3 per group). **(D)** The enzymatic activities of complex I and complex IV were determined by an ELISA-based approach (*n* = 6 per group). **(E)** Adenosine triphosphate (ATP) levels were determined in HT22 cells by an ELISA-based analysis. Relative ATP levels were plotted in IL-1β-treated HT22 cells over control HT22 cells (*n* = 8 per group). **(F)** Representative images of reactive oxygen species (ROS) (green) in HT22 cells treated with or without IL-1β using a DCFHDA fluorescence probe. Scale bar: 50 μm. Relative immunofluorescence intensity was plotted in IL-1β-treated HT22 cells over control HT22 cells (*n* = 3 per group). *P < 0.05; **P < 0.01; ***P < 0.001 by independent samples Student’s *t*-test; error bars denote the SEM.

**FIGURE 6:**
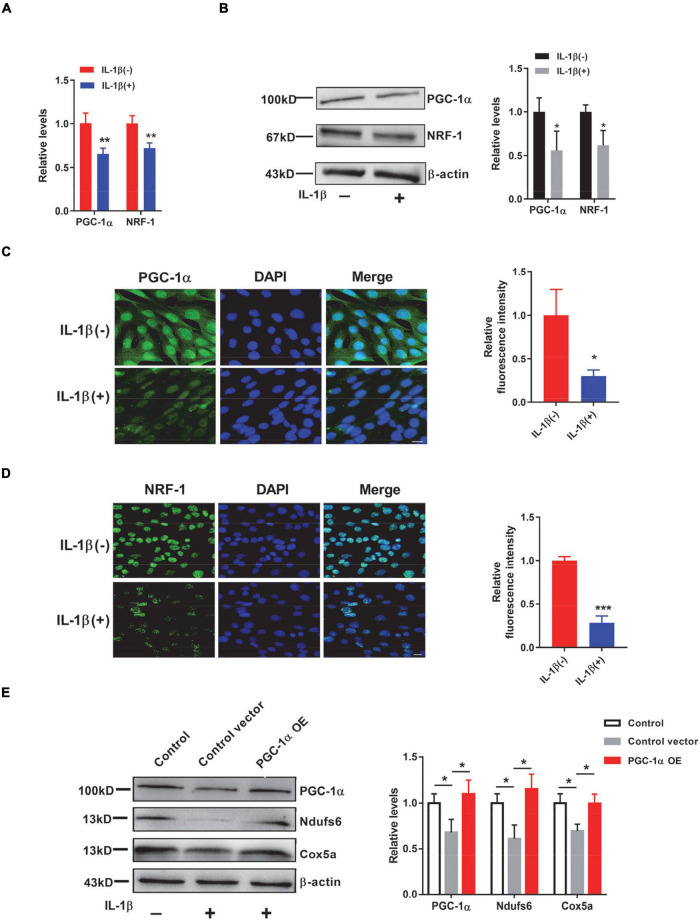
IL-1β induces reductions in peroxisome proliferator-activated receptor-γ coactivator-1α (PGC-1α) and Nuclear respiratory factor-1 (NRF-1) levels in HT22 cells. **(A,B)** Levels of PGC-1α and NRF-1 were determined by quantitative PCR (qPCR) **(A)** (*n* = 4) and by immunoblotting and densiometric analysis **(B)** in control and IL-1β-treated HT22 cells (*n* = 4 per group). β-actin was included as an input control. **(C)** Representative images of PGC-1α (green) and DAPI (blue) in control HT22 cells and HT22 cells treated with IL-1β. Scale bar: 20 μm. The relative immunofluorescence intensity of PGC-1α was plotted in IL-1β-treated HT22 cells over control HT22 cells (*n* = 3 per group). **(D)** Representative images of NRF-1 (green) and DAPI (blue) in control HT22 cells and HT22 cells treated with IL-1β. Scale bar: 20 μm. Relative immunofluorescence intensity of NRF-1 in IL-1β-treated HT22 cells over control HT22 cells (*n* = 3 per group). **(E)** HT22 cells were transfected with control plasmid (control vector) or PGC-1α overexpression plasmid (PGC-1α OE) prior to IL-1β treatment. Levels of PGC-1α, Ndufs6, and Cox5a in HT22 cells with (control vector and PGC-1α OE) or without (control) IL-1β treatment were determined by immunoblotting and densiometric analysis (*n* = 3). β-actin was included as an input control. *P < 0.05; **P < 0.01; ***P < 0.001 by independent samples Student’s *t*-test or one-way ANOVA; error bars denote the SEM.

To further investigate whether surgery-induced mitochondrial dysfunction is reproducible *in vitro*, we next treated HT22 cells with interleukin-1β (IL-1β), a potent proinflammatory cytokine, to mimic the inflammation induced by surgery *in vivo*. We first assessed the levels of respiratory chain complexes such as Atp5d, Atp5k, Cox5a, and Ndufs6. We observed that both the transcript and protein levels of these respiratory complex components were significantly decreased in the presence of IL-1β (Figures 5A,B). Immunostaining results also supported these conclusions (Figure 5C). Consequently, the activities of both complex I and complex IV were significantly decreased in the presence of IL-1β (Figure 5D). Adenosine triphosphate production was significantly decreased (Figure 5E), whereas the levels of ROS were significantly increased in HT22 cells treated with IL-1β (Figure 5F). Importantly, we also demonstrated that both the transcript and protein levels of NRF-1 and PGC-1α were significantly downregulated in the IL-1β-treated HT22 cells (Figures 6A,B). Consistently, the immunostaining results also supported these notions (Figures 6C,D). To investigate whether these IL-1β-induced changes were indeed mediated *via* PGC-1α, we overexpressed PGC-1α in HT22 cells prior to IL-1β treatment and observed that PGC-1α significantly restored the IL-1β-induced reduction in the levels of mitochondrial complex subunits (Figure 6E). Taken together, *in vivo* and *in vitro*, we demonstrated that PND-induced neuroinflammation results in reductions in respiratory complex components and impaired mitochondrial respiratory functions.

## Discussion

In this study, we investigated PND using a mouse model of orthopedic surgery, which produces neuroinflammation (Terrando et al., 2011; Xiong et al., 2018) and is, therefore, regarded as a common etiology for PND (Saxena et al., 2019; Yang et al., 2020). We conducted open reduction and intramedullary nailing in mice, which is a more common surgical procedure than splenectomy models in the clinic and is a well-recognized mouse model in the field (Eckenhoff et al., 2020). Wang et al. (2020) demonstrated that sevoflurane anesthesia-induced PND leads to dysfunction in mitochondria and oxidative stress-related signaling pathways in the hippocampus of aged rats. Our model closely mimics clinical practice, as no clinical practice involves anesthesia without further surgical procedures. In this study, we illustrated that the transcript levels of mitochondrial respiratory chain subunits appeared to decrease as early as 1 day post-surgery, whereas the protein levels exhibited a strong decrease at 3 days post-surgery. Neuroinflammation is a widely accepted mechanism of PND. In this study, we also identified a significant increase in the inflammatory factors IL-6 and IL-1β in the hippocampus of PND mice, confirming that neuroinflammation indeed contributes to PND. In addition to neuroinflammation, we also defined another etiology of PND: a decline in mitochondrial function contributes substantially to PND. These two mechanisms do not seem to conflict with each other.

The hippocampus is highly involved in cognitive function in rodents and humans (Bird and Burgess, 2008; Li et al., 2021); therefore, we focused on this brain region for further transcriptome sequencing. We observed significantly reduced transcript levels of multiple respiratory complex genes in the hippocampus of surgical mice at 1 day post-surgery. These results are not supported by the previous report, wherein no mitochondrial function-related changes were observed in the mice subjected to the same surgical procedure (Xiang et al., 2019). This inconsistency likely comes from the age difference, as aging is considered a major risk factor for PND. Xiang et al. used 12-week-old (relatively young) mice in their study (Xiang et al., 2019), whereas we used approximately 8-month-old (relatively old) mice. In addition to the age issue, the timing of tissue collection also matters, as we collected hippocampal tissues at 1 day post-surgery, whereas Xiang et al. performed tissue collection at 6 h post-surgery.

This surgical procedure that induced a decrease in the mRNA levels of the respiratory complexes is not accompanied by any decrease in the protein levels of the same respiratory complexes but by increased protein levels of IL-6 and IL-1β. The protein changes in these respiratory complexes appear at a later time point (3 days post-surgery). These findings demonstrate that these two identified mechanisms of PND occur in a sequential manner, as inflammation occurs preceding mitochondrial dysfunction. In support of these findings, mitochondrial functions, assessed by ATP and ROS production, remained unaltered in the hippocampus of PND mice at 1 day post-surgery, and a significant reduction in mitochondrial function was observed in the same brain region of PND mice at 3 days post-surgery. This further supports that changes in mitochondrial function coincide with changes in respiratory chain complexes.

The expression of mitochondrial respiratory chain complexes is reportedly controlled by nuclear respiratory factors (NRFs) such as NRF-1 and NRF-2. In particular, NRF-1 is known to be involved in the transcription of genes encoding subunits of mitochondrial respiratory chain complexes (Bouchez and Devin, 2019). Nuclear respiratory factor-1 depletion leads to early mouse embryonic lethality caused by severe mitochondrial DNA depletion (Diman et al., 2016). In this study, we observed that NRF-1 levels were significantly decreased in the hippocampus of surgical mice at 1 day post-surgery, supporting that level changes in respiratory complexes are due to changes in NRF-1. The PGC-1 family, consisting of PGC-1α, PGC-1β, and PRC, plays a central role in governing the transcriptional control of mitochondrial biogenesis and respiratory function (Scarpulla, 2011). Among them, PGC-1α is a “master regulator” in mitochondrial biogenesis by inducing the transcription of NRF (Tran et al., 2011; Wang et al., 2015). In this study, we observed that PGC-1α levels were significantly reduced in the hippocampus of surgical mice at 1 day post-surgery.

Importantly, PGC-1α levels were substantially downregulated in response to the inflammatory response (Tran et al., 2011; Rius-Perez et al., 2020). For example, IL-6 levels in the muscles are negatively correlated with PGC-1α levels (Handschin et al., 2007). The inflammatory-induced PGC-1α decrease is likely due to the activation of the NF-κB pathway (Eisele and Handschin, 2014). It has been reported that improved mitochondrial function by elamipretide (SS-31) could improve PND in a mouse/rat model (Wu et al., 2017; Zhao et al., 2019; Zuo et al., 2020), suggesting that mitochondrial function is highly related to PND. In this study, we clearly demonstrate that PND-induced mitochondrial dysfunction occurs due to decreased respiratory complex levels resulting from increased proinflammatory cytokines and decreased transcription factors PGC-1α and NRF-1. ROS mainly come from the mitochondrial respiratory chain, and there is strong evidence showing that mitochondrial ROS production plays a critical role in damaging CCs and initiating cell death (Di Meo et al., 2016). Reactive oxygen species may induce neuronal apoptosis and lead to cognitive deficits (Berg et al., 2011). Oxidative stress is a byproduct of surgery and anesthesia (Skvarc et al., 2018). In this study, surgical mice at 3 days post-surgery showed a substantial increase in ROS production in the hippocampus. Meanwhile, the MMP was also decreased. These findings suggest that mitochondrial dysfunction-induced ROS production contributes to PND.

The HT22 cell line is derived from mouse hippocampal neurons and is widely used to simulate primary hippocampal neurons (Reddy et al., 2018). The inflammatory cytokine IL-1β was used to induce inflammation *in vitro*. Our *in vitro* findings support the conclusion obtained from *in vivo* animal models. Taken together, PND-induced neuroinflammation suppresses the levels of the nuclear mitochondrial transcription factors PGC-1α and NRF-1, consequently reducing the levels of respiratory complexes and ultimately reducing mitochondrial functions. Therefore, we propose a novel mechanism for PND involving mitochondrial dysfunction and potentially provide a novel therapeutic target for the prevention and treatment of PND.

## Data Availability Statement

The datasets presented in this study can be found in online repositories. The name of the repository and accession number can be found below: https://www.ncbi.nlm.nih.gov/geo/query/acc.cgi?, accession number: GSE178995.

## Ethics Statement

The animal study was reviewed and approved by Animal Studies Committee at University of Science and Technology.

## Author Contributions

KH, JZ, XC, and QL conceived and designed the study and wrote the manuscript. KH, DL, XW, and WZ performed the *in vivo* experiments and analyzed the data. KH, XM, and XW performed the *in vitro* experiments. SW and WZ conducted the data analysis. All authors contributed to the article and approved the submitted version.

## Conflict of Interest

The authors declare that the research was conducted in the absence of any commercial or financial relationships that could be construed as a potential conflict of interest.

## Publisher’s Note

All claims expressed in this article are solely those of the authors and do not necessarily represent those of their affiliated organizations, or those of the publisher, the editors and the reviewers. Any product that may be evaluated in this article, or claim that may be made by its manufacturer, is not guaranteed or endorsed by the publisher.

## Supplementary Material

Supplementary Figure 1 Control and surgery (6 days post-surgery) mice were subjected to the open field test (*n* = 12 per group). **(A)** Total distance traveled and the time spent in the central zone of the arena were recorded. **(C)** Brain–blood-barrier permeability was determined in naïve (no treatment), control (anesthesia only), and surgery (1 day post-surgery) mice by an Evans blue injection-based approach (*n* = 6 per group). ^***^*P* < 0.001 by independent samples Student’s *t*-test; n.s. not significant; error bars denote the SEM.

## Abbreviations

PND: perioperative neurocognitive disorders
POCD: postoperative cognitive dysfunction
POD: postoperative delirium
CSF: cerebrospinal fluid
NADH: nicotinamide adenine dinucleotide
ATP: adenosine triphosphate
MMP: mitochondrial membrane potential
DCFHDA: 2′,7′-dichlorofluorescein diacetate
ROS: reactive oxygen species
FC: fear conditioning
DMEM: Dulbecco’s Modified Eagle Medium
FBS: fetal bovine serum
NOR: novel object recognition
IL-6: interleukin-6
IL-1β: interleukin-1β
DEGs: differentially expressed genes
KEGG: Kyoto Encyclopedia of Genes and Genomes
GO: gene ontology
BP: biological process
MF: molecular function
CC: cellular component
PGC-1α: peroxisome proliferator-activated receptor-γ coactivator-1α
NRF-1: nuclear respiratory factor-1.
