# Supplementary figures and images for: Hippocampus-Based Mitochondrial Respiratory Function Decline Is Responsible for Perioperative Neurocognitive Disorders
Source: Front Aging Neurosci. 2022 Feb 9;14:772066. doi: 10.3389/fnagi.2022.772066 (PMC8865419; doi:10.3389/fnagi.2022.772066)

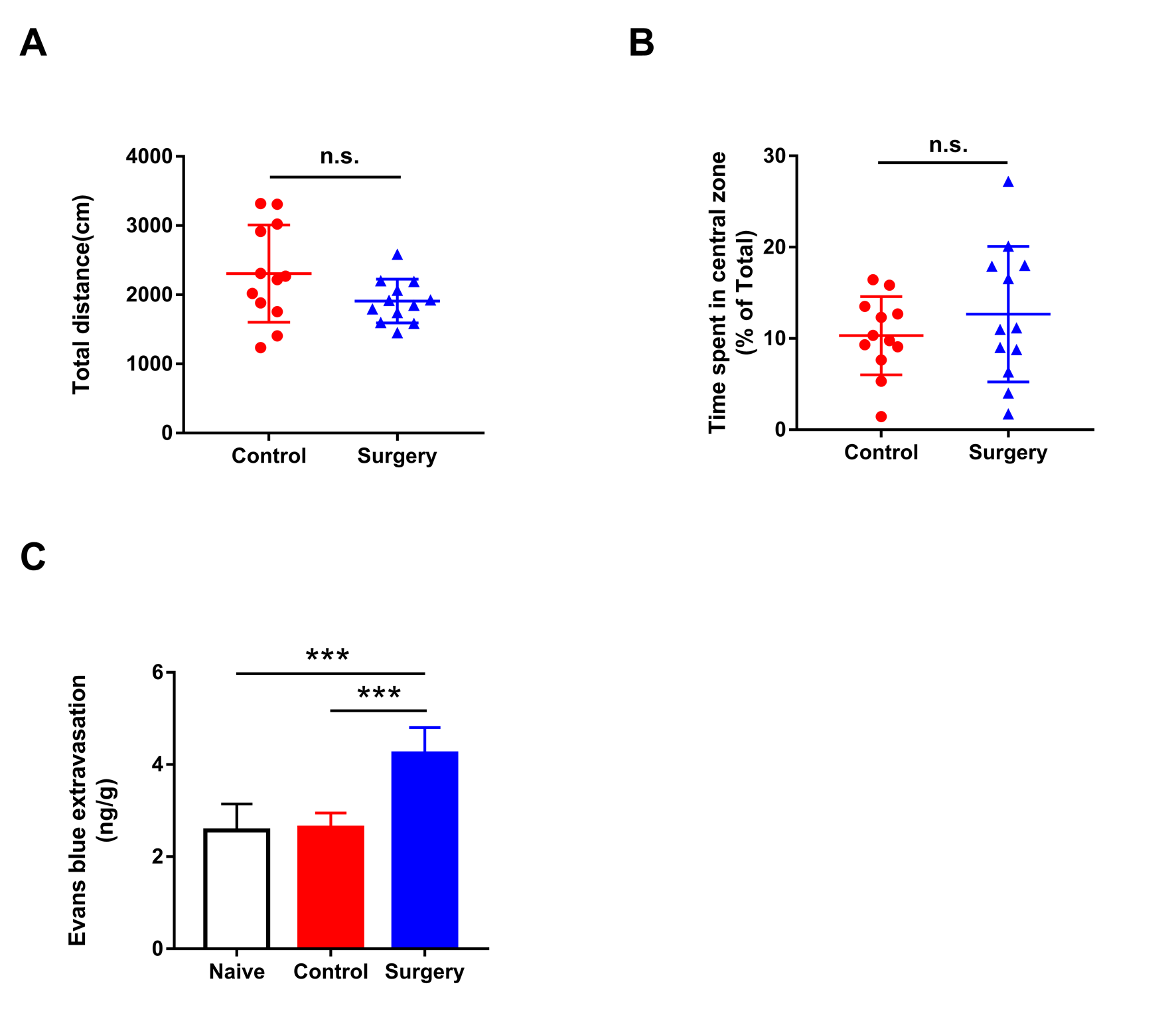

Supplement: Supplementary Figure 1 — Control and surgery (6 days post-surgery) mice were subjected to the open field test (n = 12 per group). (A) Total distance traveled and the time spent in the central zone of the arena were recorded. (C) Brain–blood-barrier permeability was determined in naïve (no treatment), control (anesthesia only), and surgery (1 day post-surgery) mice by an Evans blue injection-based approach (n = 6 per group). ***P < 0.001 by independent samples Student’s t-test; n.s. not significant; error bars denote the SEM. [file Image_1.TIF]

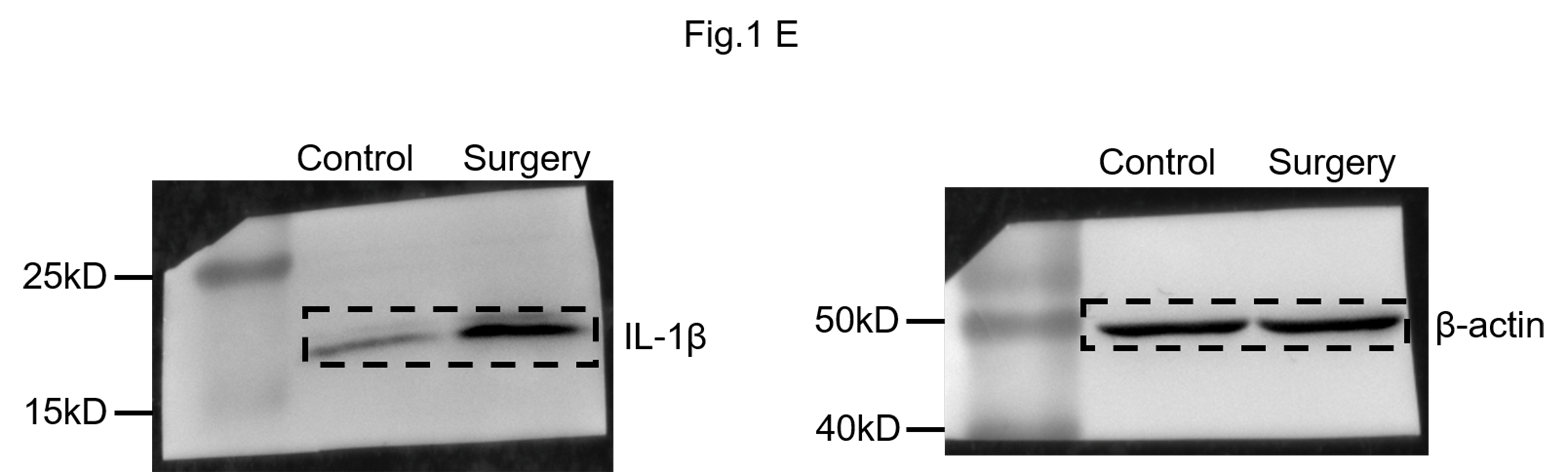

Supplement: Supplementary file 2 [file Data_Sheet_1.zip › Fig.1/Fig.1E WB/Fig1E.tif]

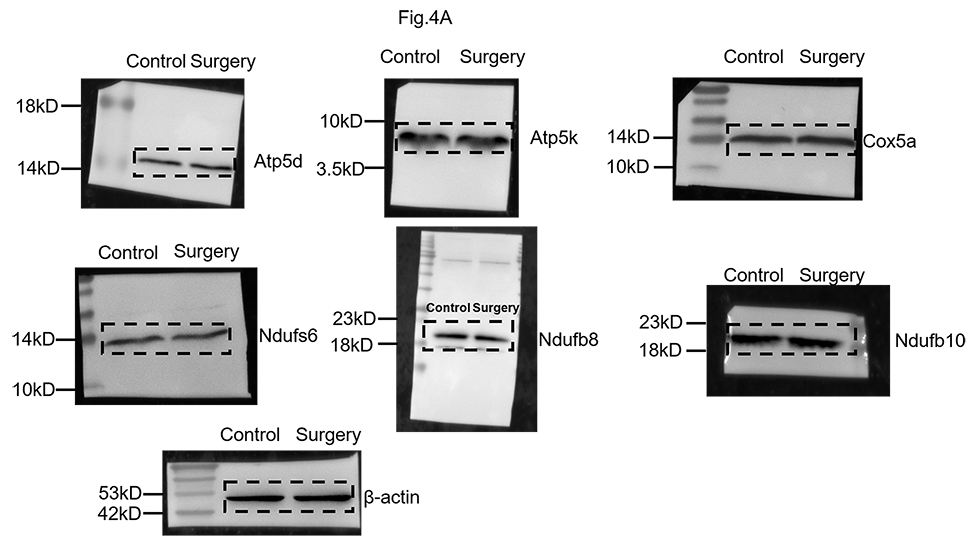

Supplement: Supplementary file 3 [file Data_Sheet_2.zip › Fig.4/Fig.4A,B/Fig.4A.tif]

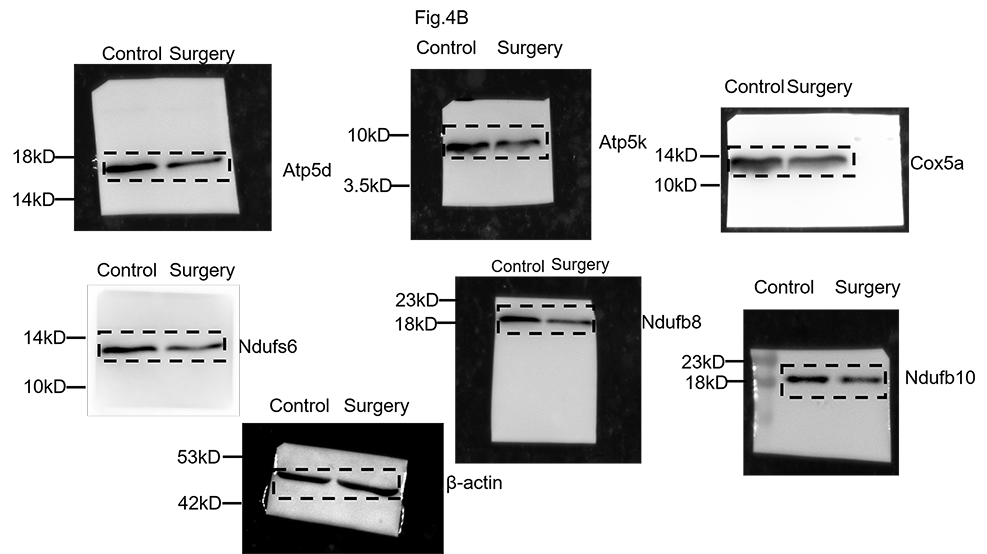

Supplement: Supplementary file 3 [file Data_Sheet_2.zip › Fig.4/Fig.4A,B/Fig.4B.tif]

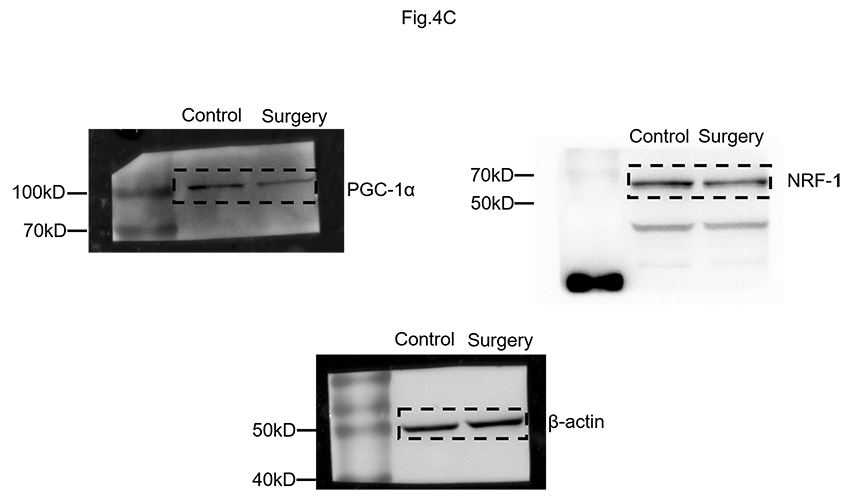

Supplement: Supplementary file 3 [file Data_Sheet_2.zip › Fig.4/Fig.4C/Fig.4C.tif]

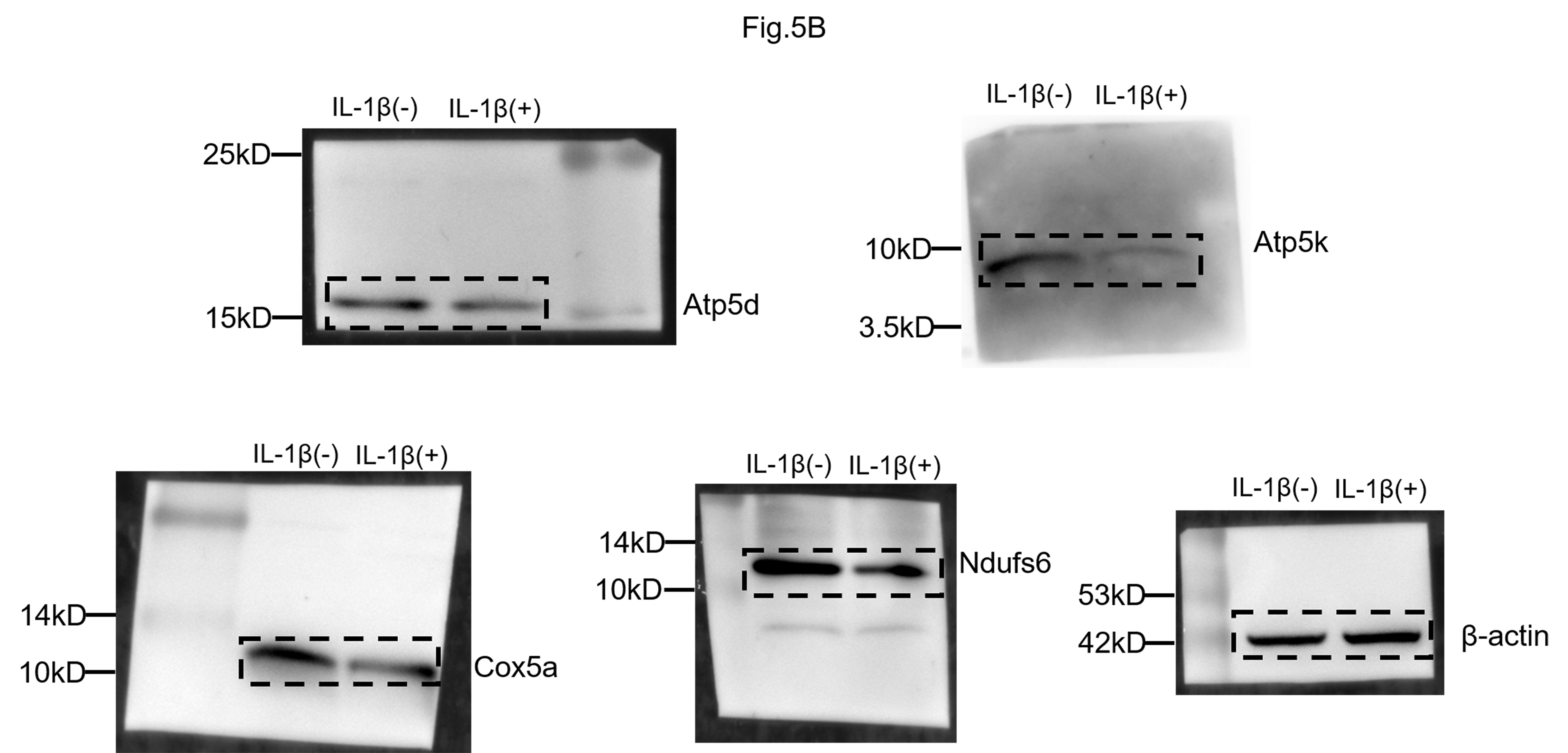

Supplement: Supplementary file 3 [file Data_Sheet_2.zip › Fig.5/Fig.5B/Fig.5B.jpg]

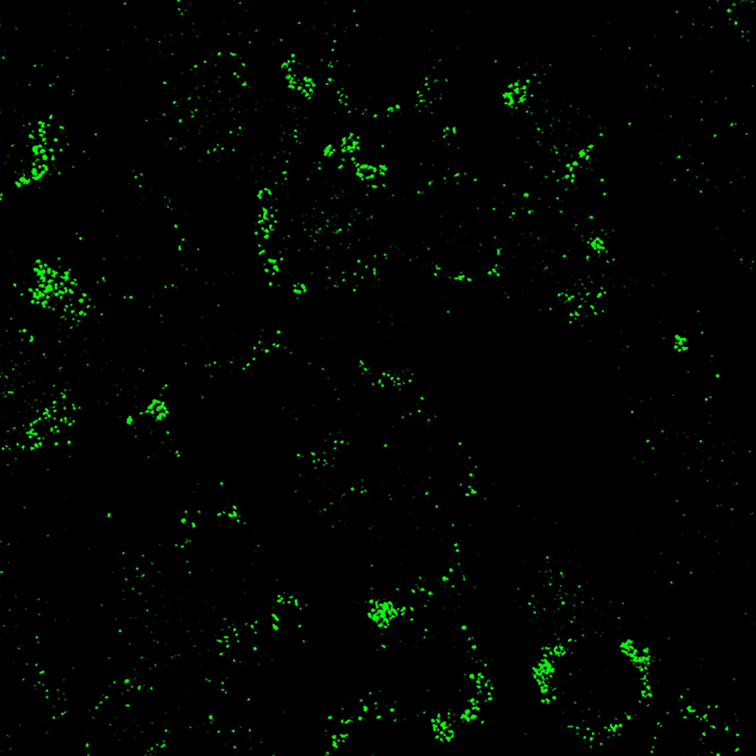

Supplement: Supplementary file 3 [file Data_Sheet_2.zip › Fig.5/Fig.5C/C/Ndufs6 C .jpg]

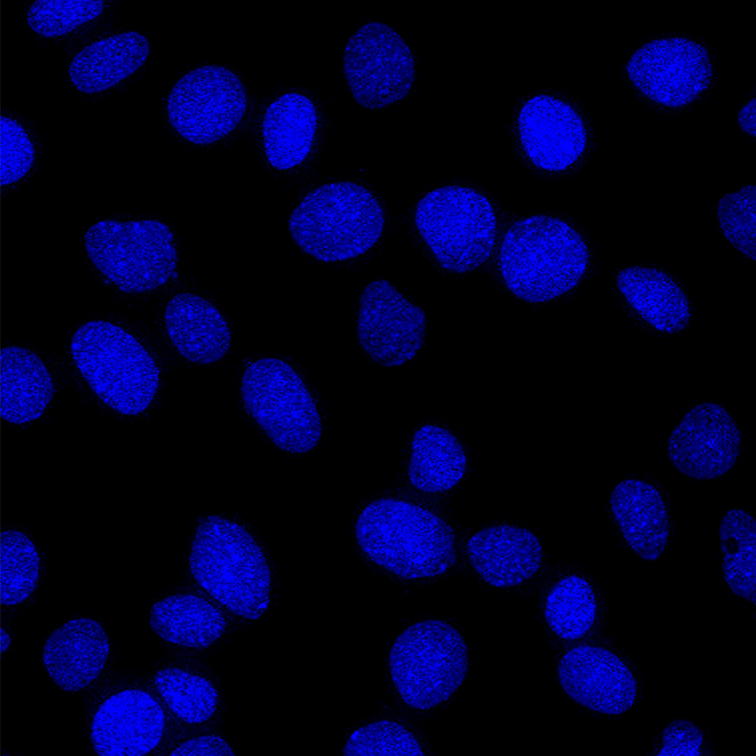

Supplement: Supplementary file 3 [file Data_Sheet_2.zip › Fig.5/Fig.5C/C/Ndufs6 C DAPI.jpg]

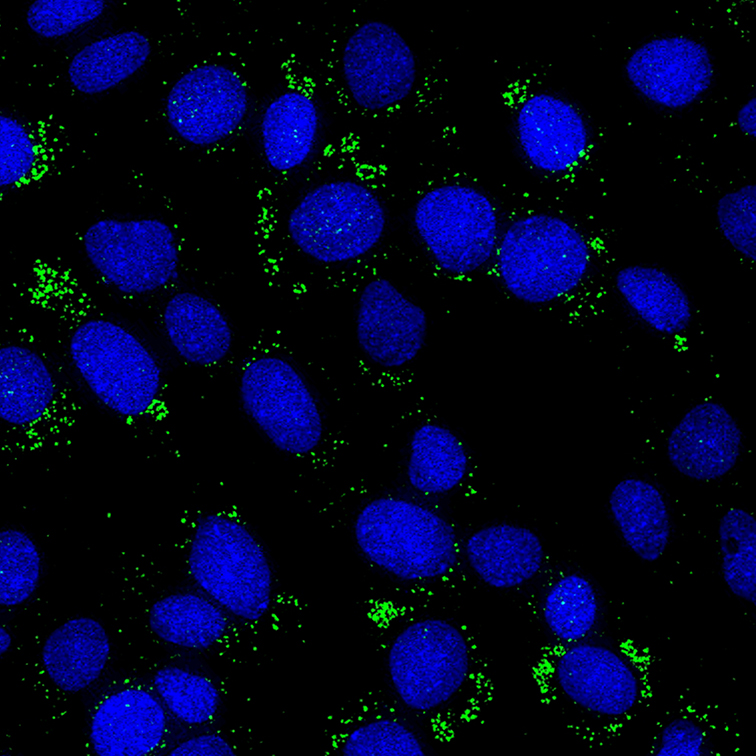

Supplement: Supplementary file 3 [file Data_Sheet_2.zip › Fig.5/Fig.5C/C/Ndufs6 C confocal.jpg]

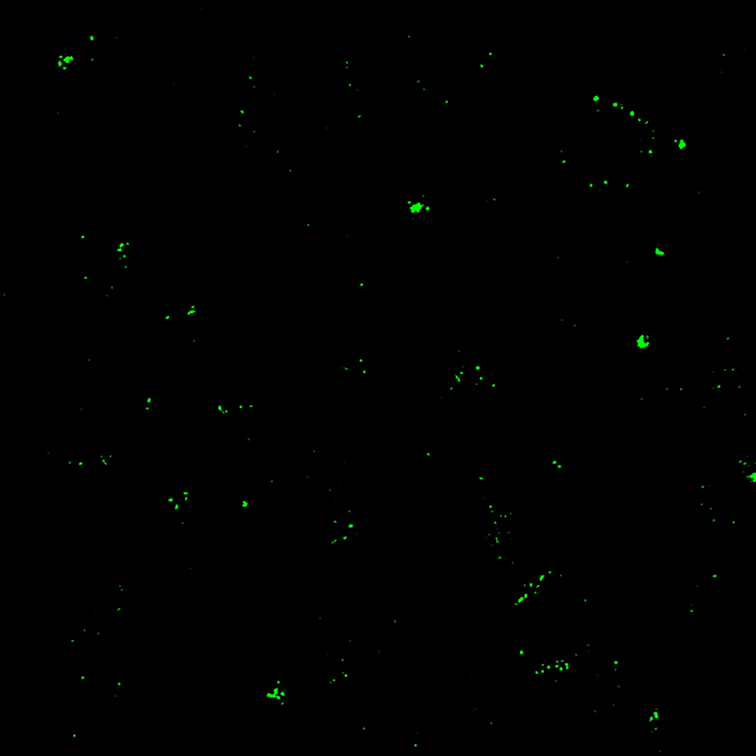

Supplement: Supplementary file 3 [file Data_Sheet_2.zip › Fig.5/Fig.5C/S/Ndufs6 S .jpg]

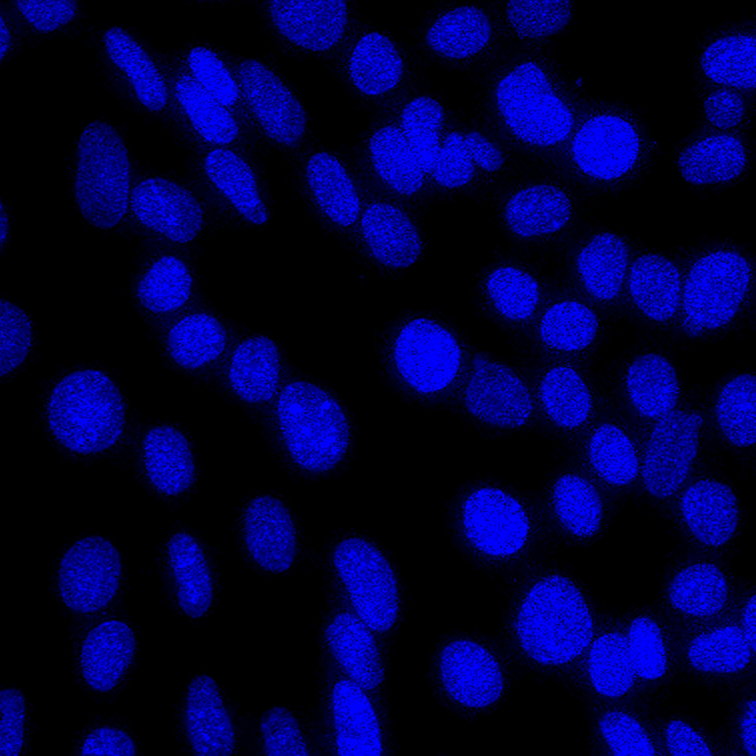

Supplement: Supplementary file 3 [file Data_Sheet_2.zip › Fig.5/Fig.5C/S/Ndufs6 S DAPI.jpg]

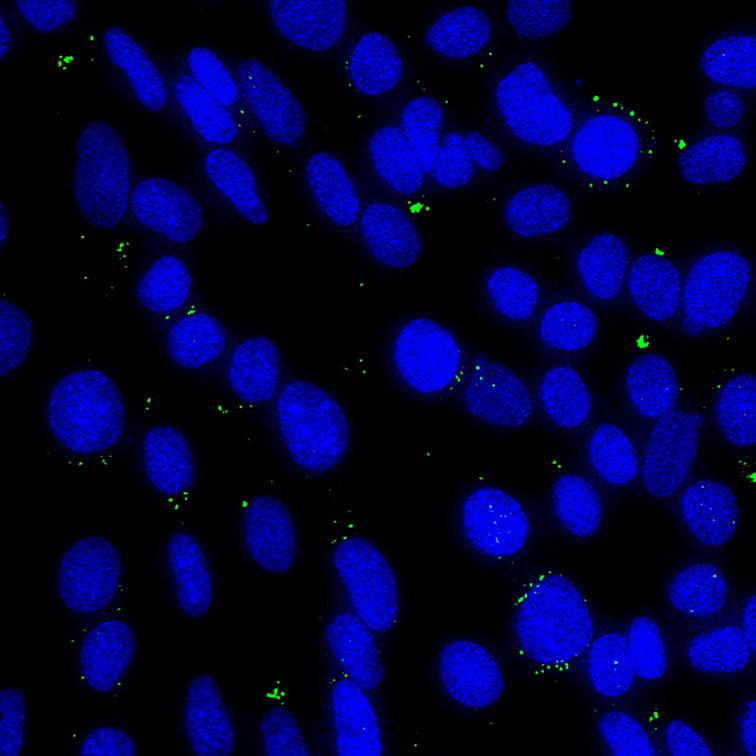

Supplement: Supplementary file 3 [file Data_Sheet_2.zip › Fig.5/Fig.5C/S/Ndufs6 S confocal.jpg]

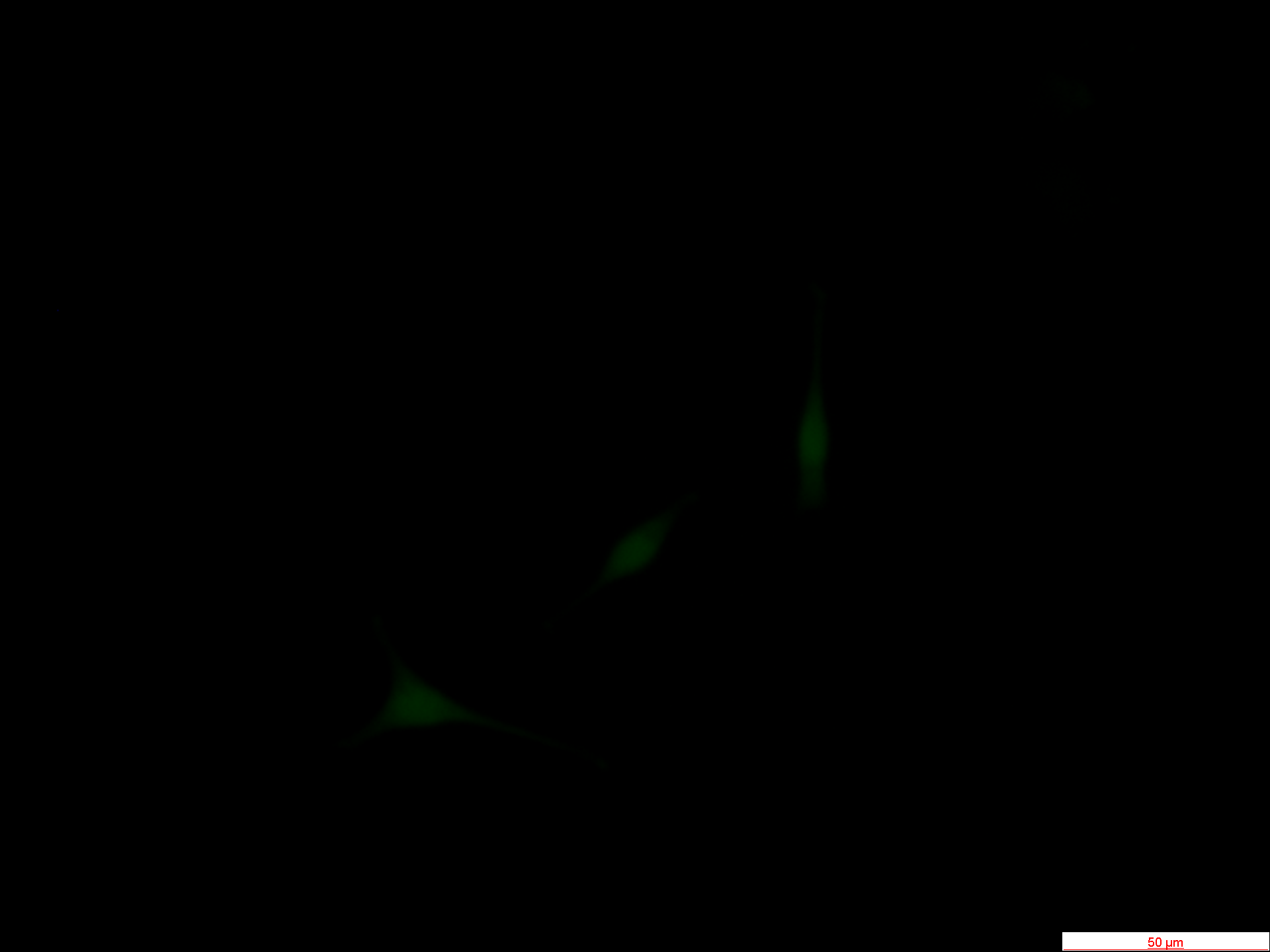

Supplement: Supplementary file 3 [file Data_Sheet_2.zip › Fig.5/Fig.5F/C/ROS C1.tif]

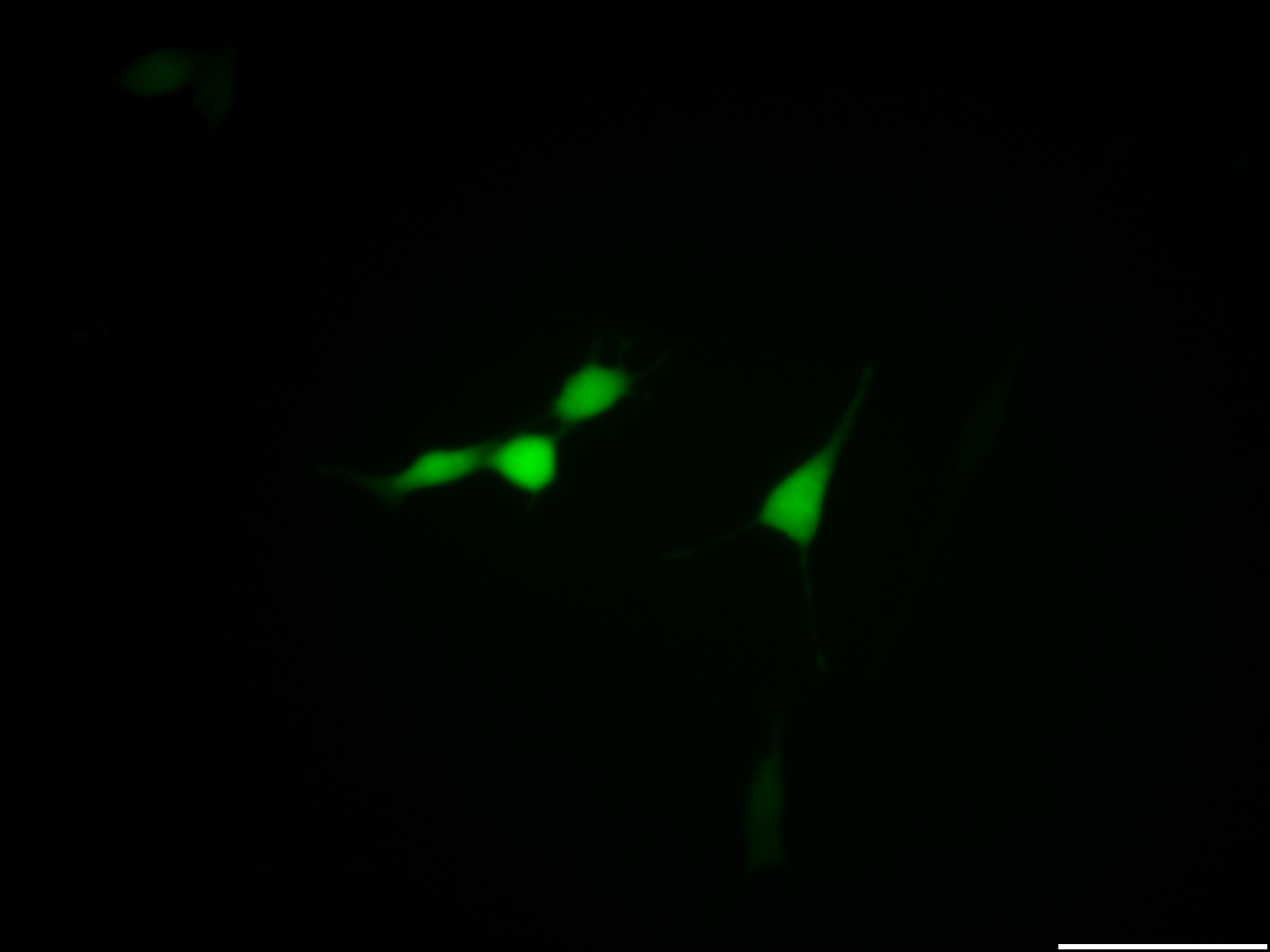

Supplement: Supplementary file 3 [file Data_Sheet_2.zip › Fig.5/Fig.5F/S/ROS S1.tif]

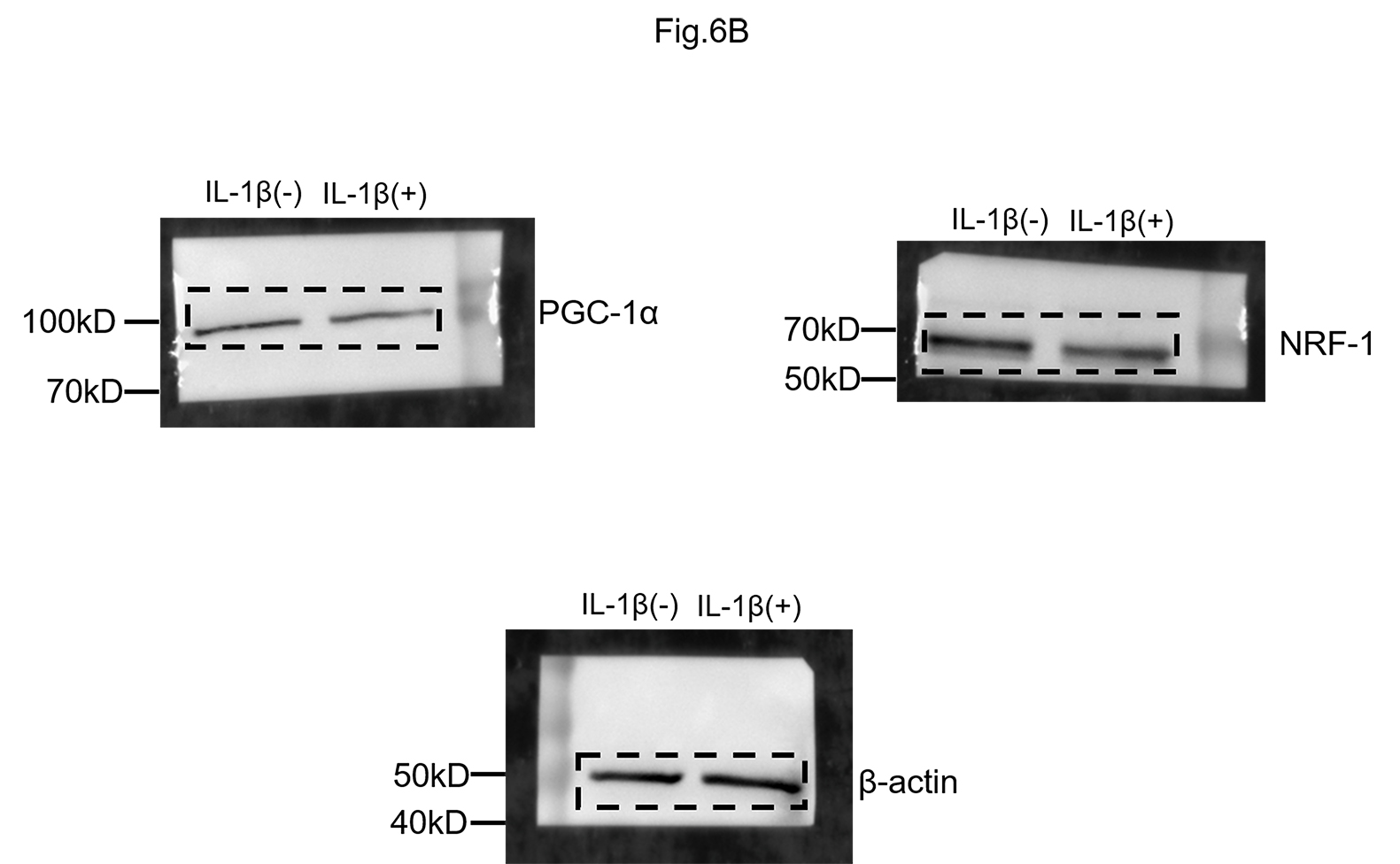

Supplement: Supplementary file 3 [file Data_Sheet_2.zip › Fig.6/Fig.6B/Fig.6B.jpg]

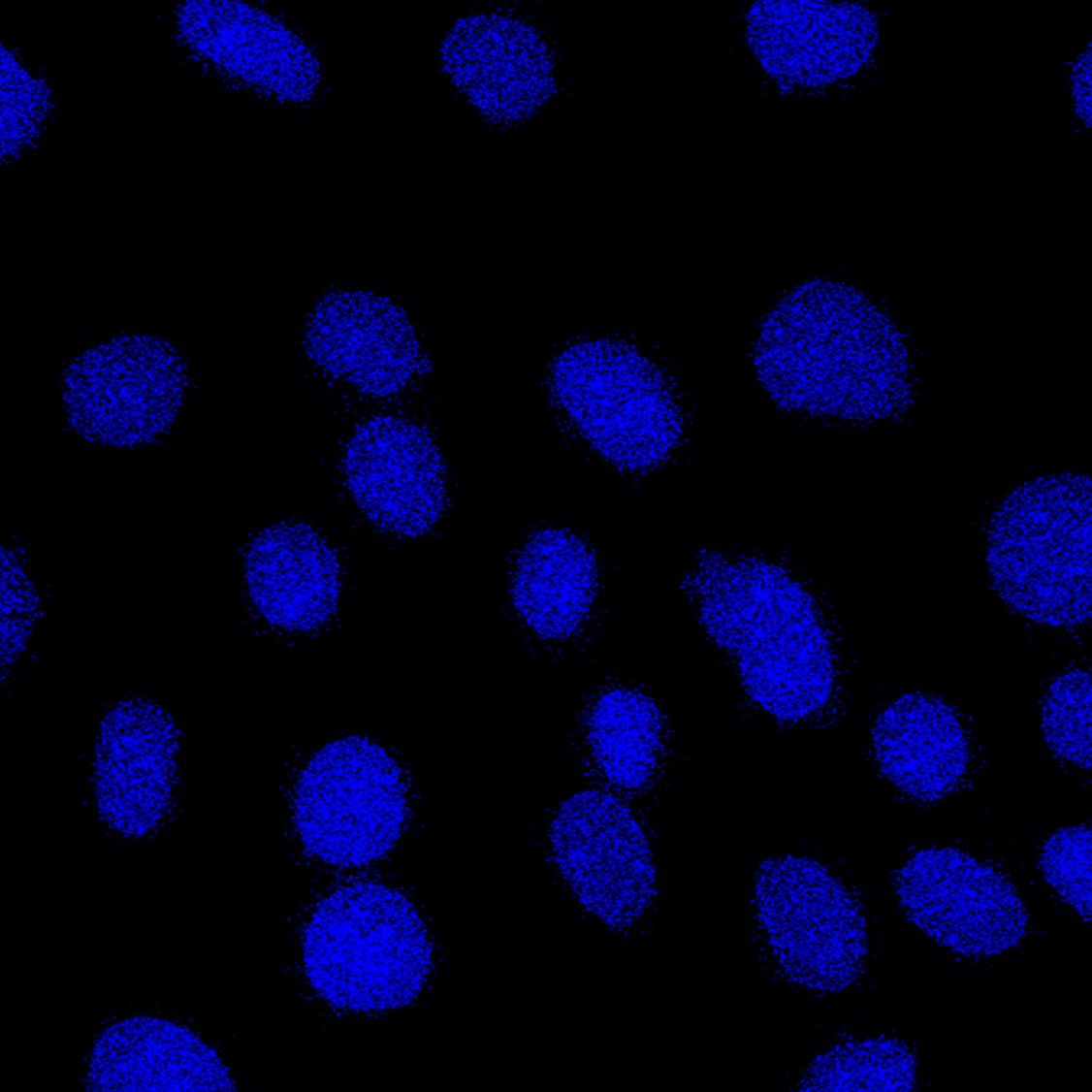

Supplement: Supplementary file 3 [file Data_Sheet_2.zip › Fig.6/Fig.6C/C/PGC-1a DAPI.jpg]

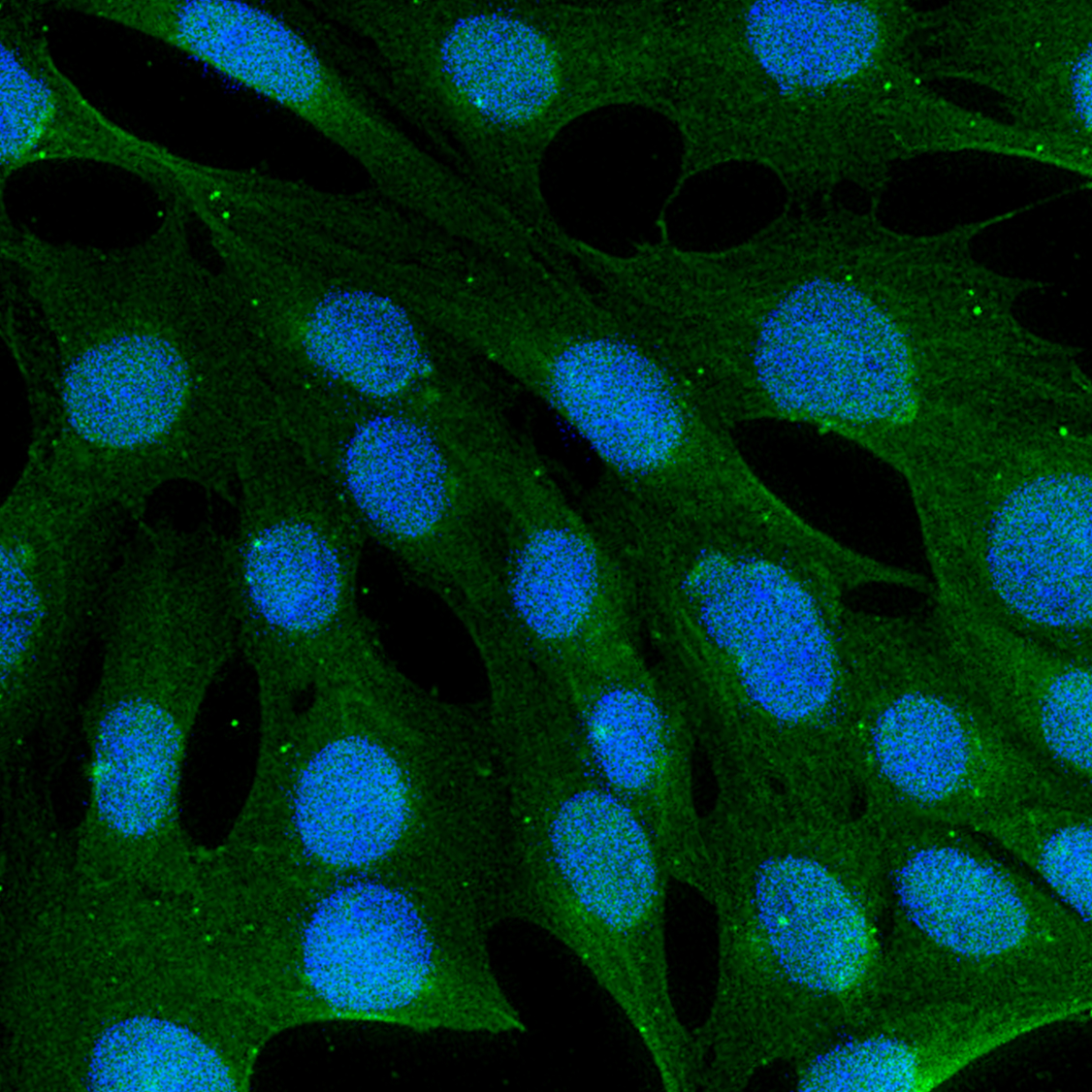

Supplement: Supplementary file 3 [file Data_Sheet_2.zip › Fig.6/Fig.6C/C/PGC-1a confocal.jpg]

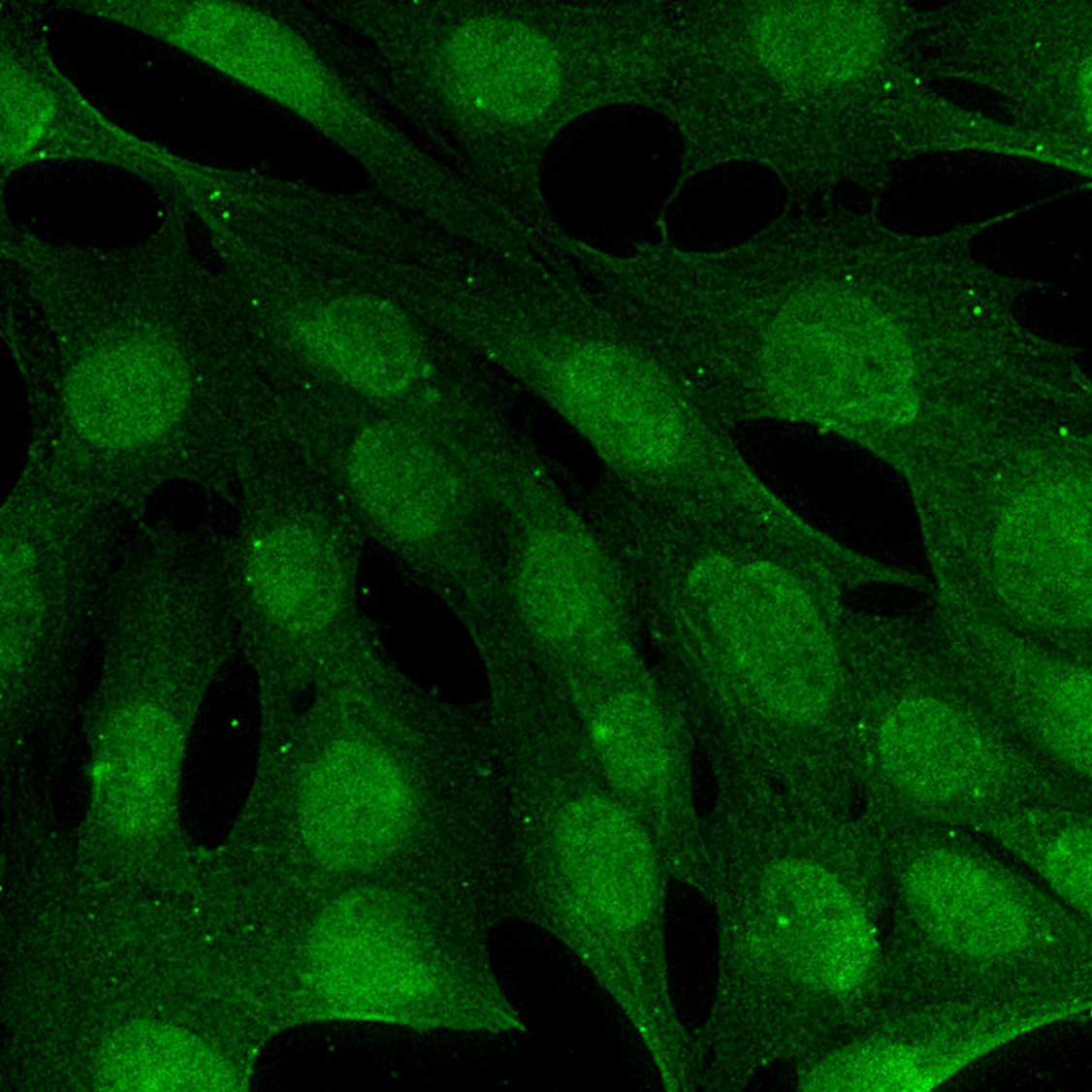

Supplement: Supplementary file 3 [file Data_Sheet_2.zip › Fig.6/Fig.6C/C/PGC-1a.jpg]

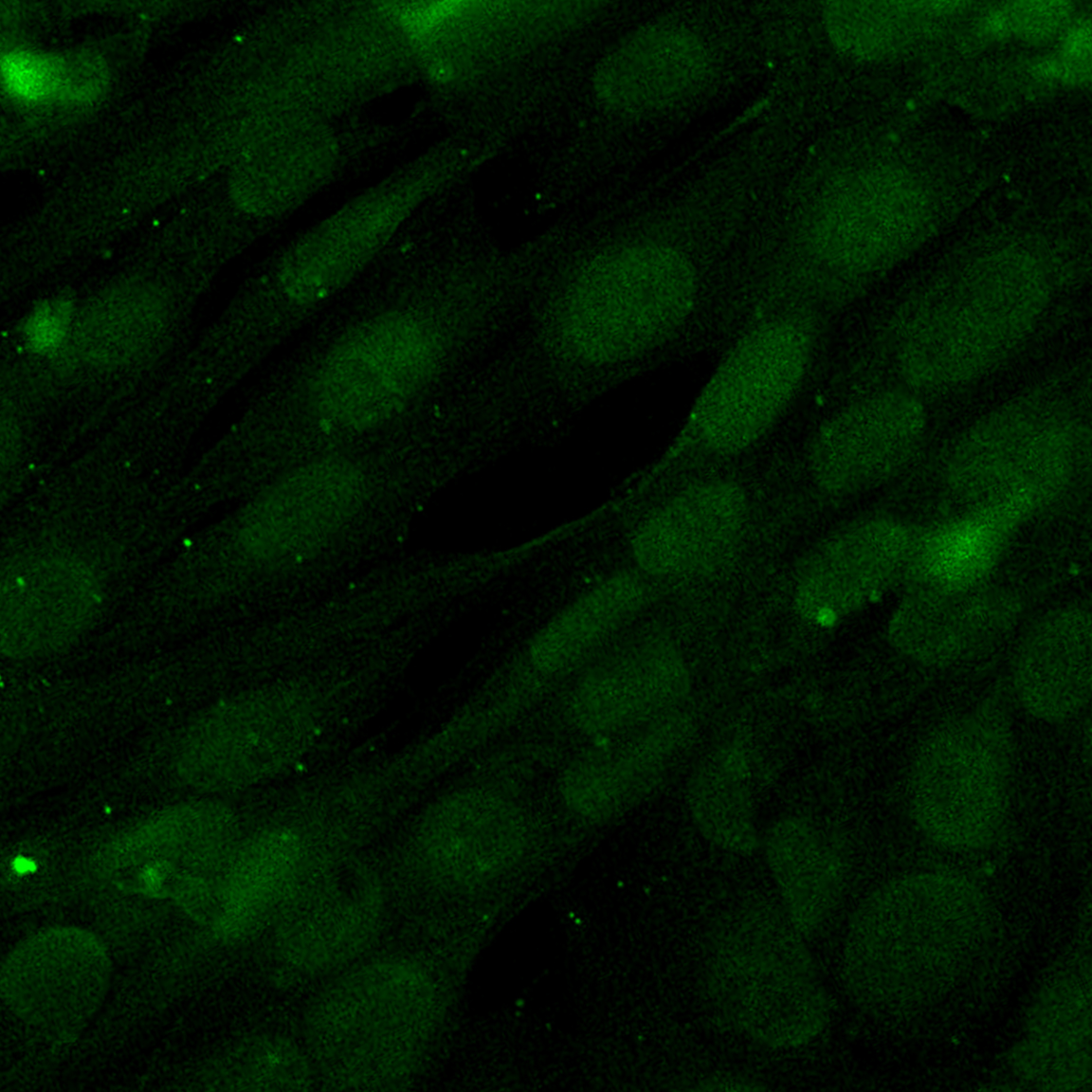

Supplement: Supplementary file 3 [file Data_Sheet_2.zip › Fig.6/Fig.6C/S/PGC-1a S .jpg]

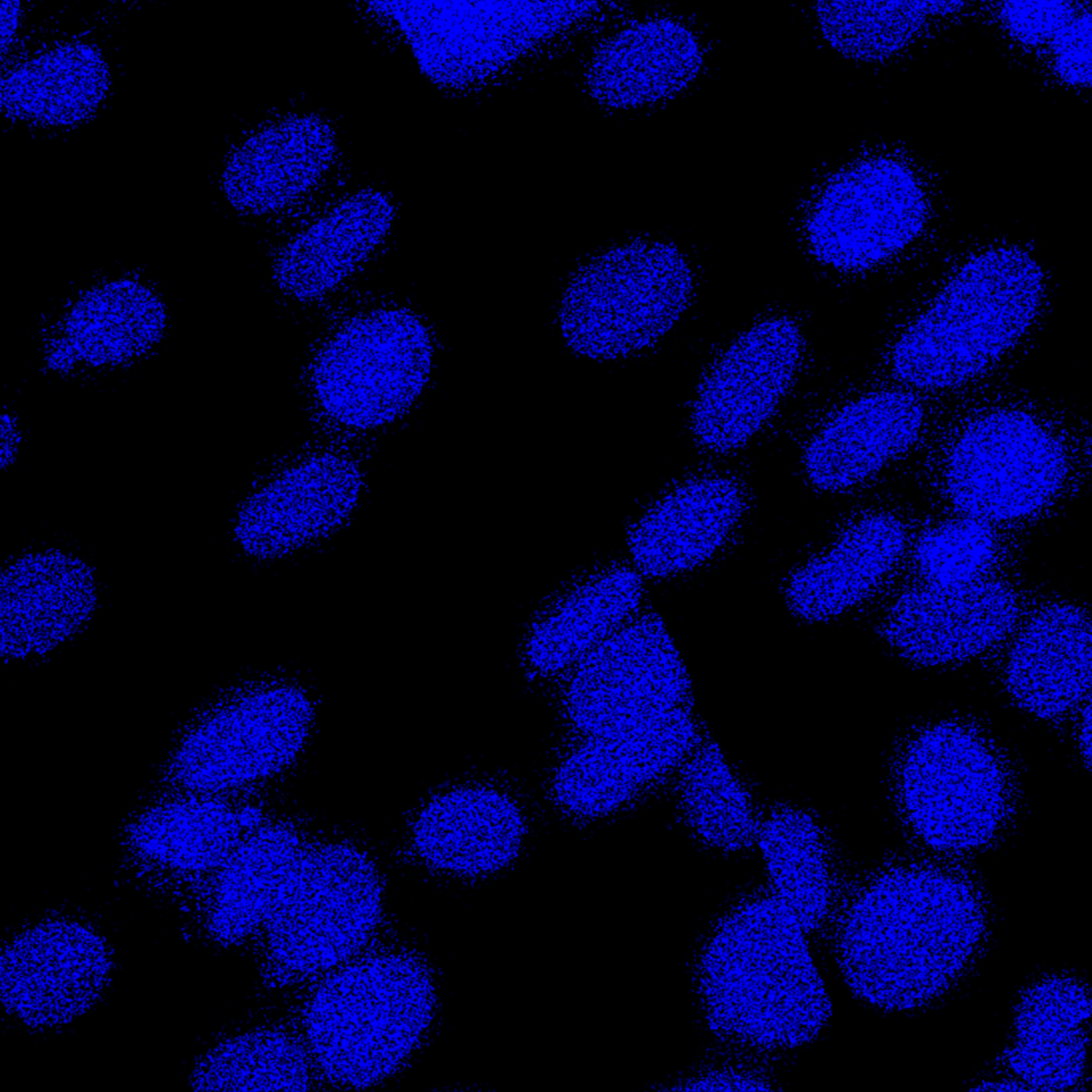

Supplement: Supplementary file 3 [file Data_Sheet_2.zip › Fig.6/Fig.6C/S/PGC-1a S DAPI.jpg]

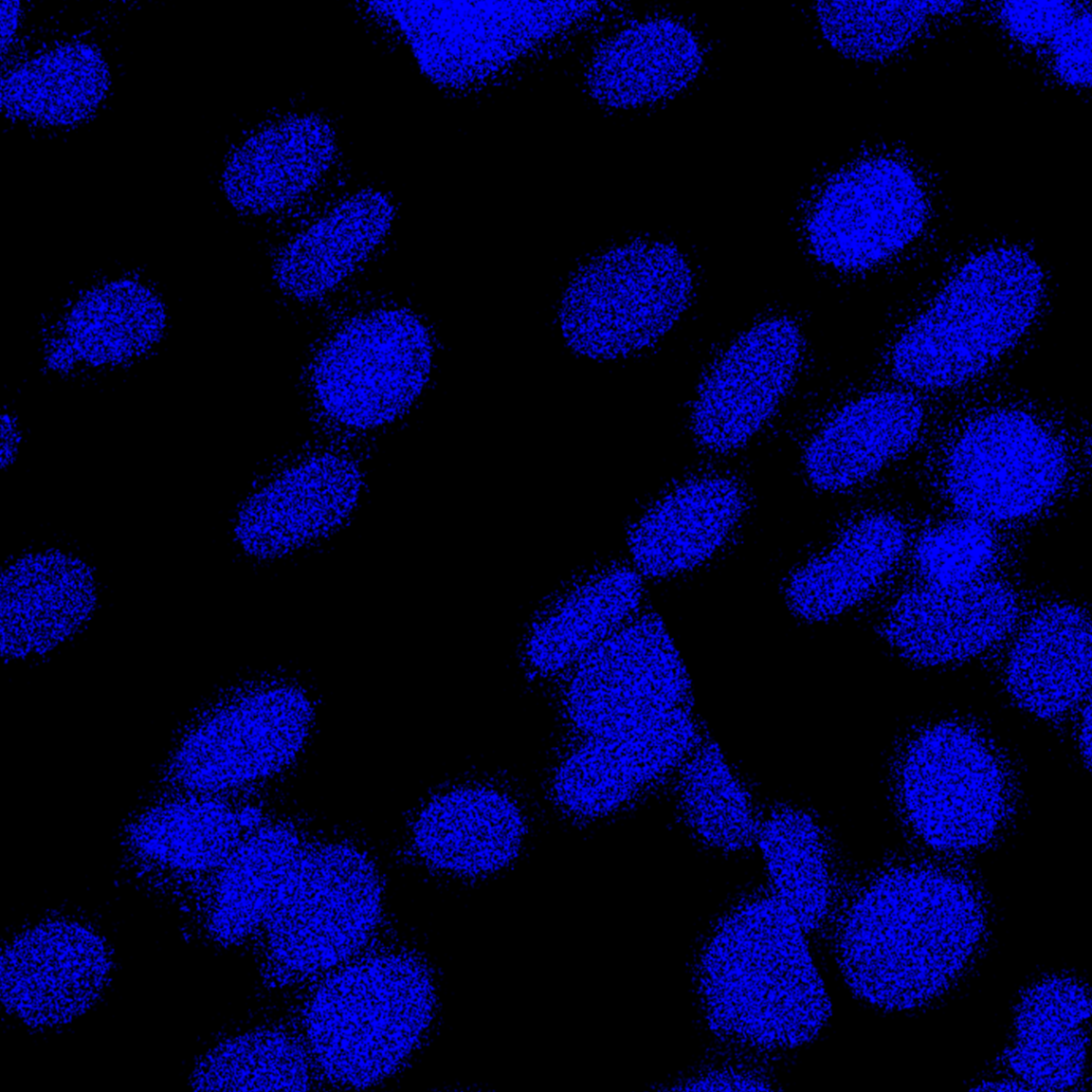

Supplement: Supplementary file 3 [file Data_Sheet_2.zip › Fig.6/Fig.6C/S/PGC-1a S DAPI.tif]

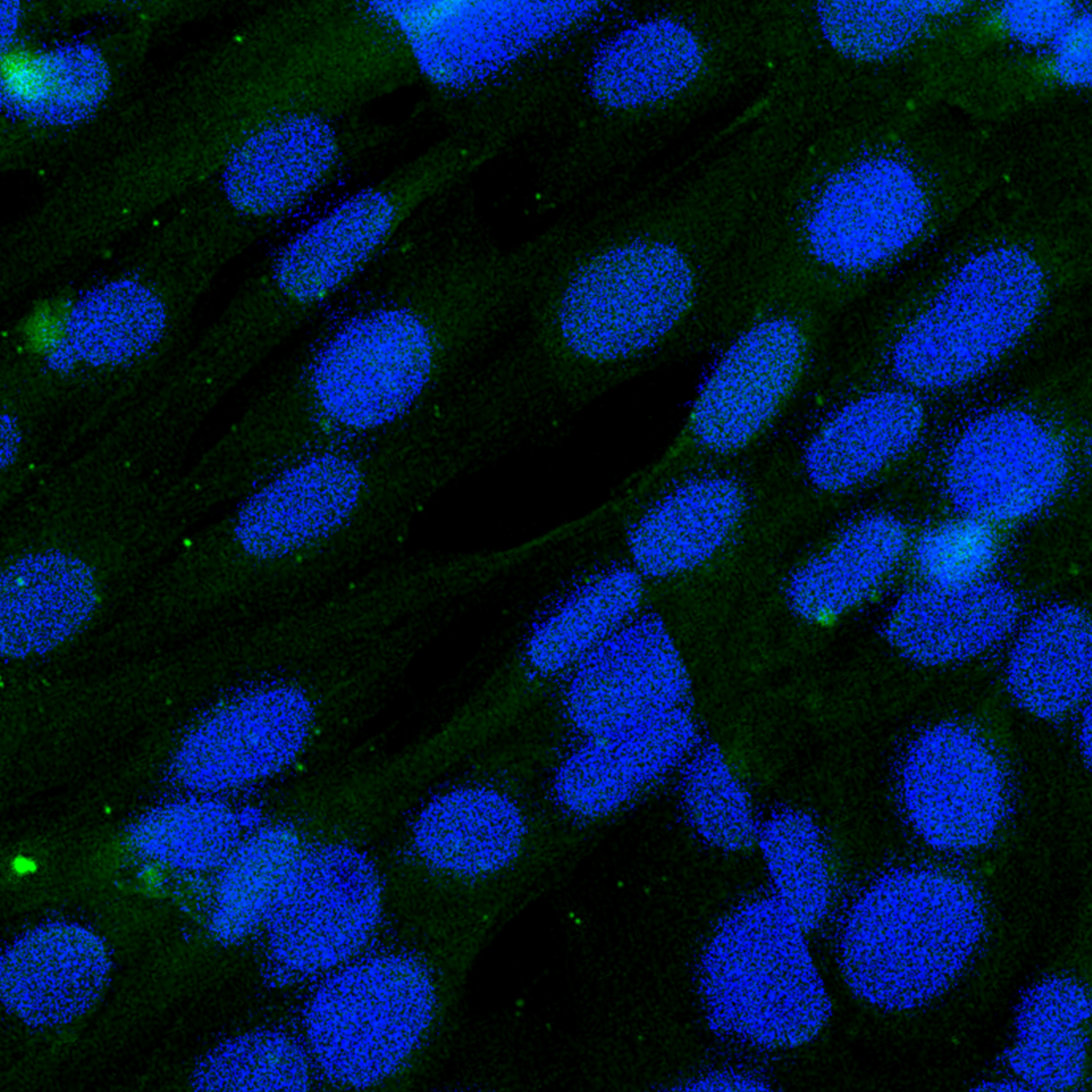

Supplement: Supplementary file 3 [file Data_Sheet_2.zip › Fig.6/Fig.6C/S/PGC-1a S confocal.jpg]

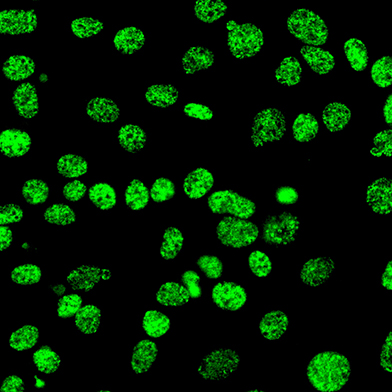

Supplement: Supplementary file 3 [file Data_Sheet_2.zip › Fig.6/Fig.6D/C/NRF-1 C .jpg]

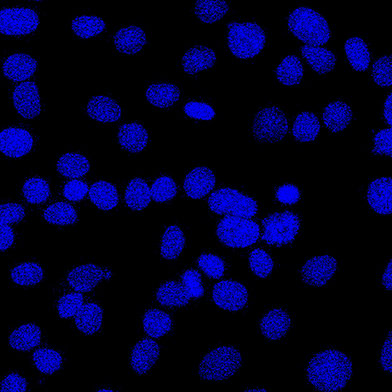

Supplement: Supplementary file 3 [file Data_Sheet_2.zip › Fig.6/Fig.6D/C/NRF-1 C DAPI.jpg]

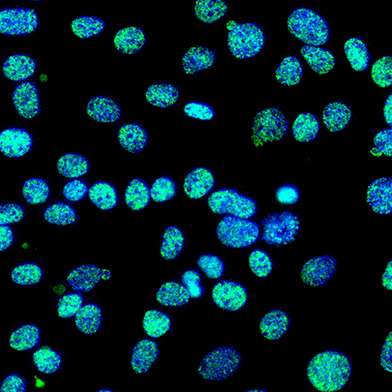

Supplement: Supplementary file 3 [file Data_Sheet_2.zip › Fig.6/Fig.6D/C/NRF-1 C confocal.jpg]

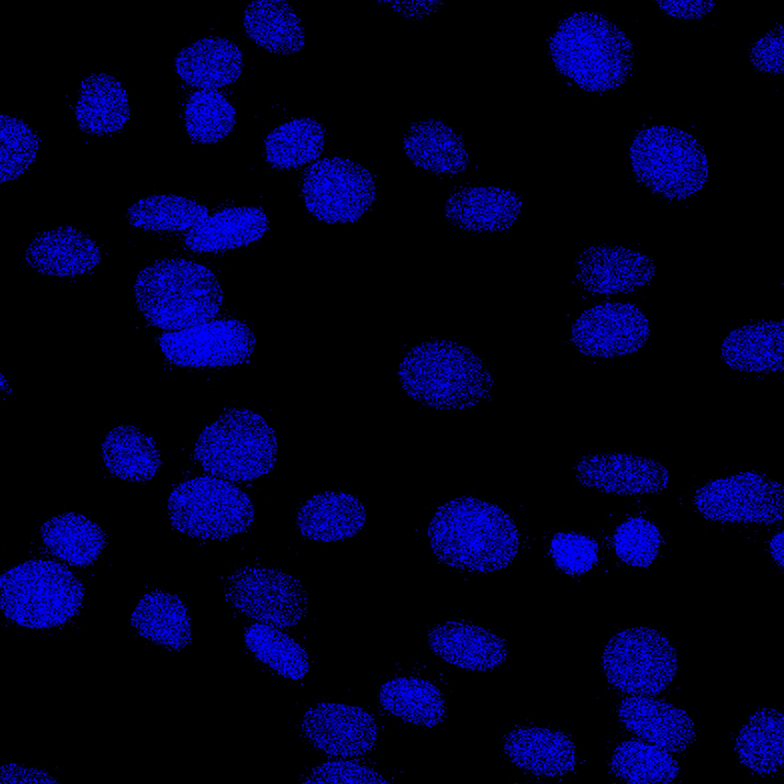

Supplement: Supplementary file 3 [file Data_Sheet_2.zip › Fig.6/Fig.6D/S/NRF-1 S DAPI.jpg]

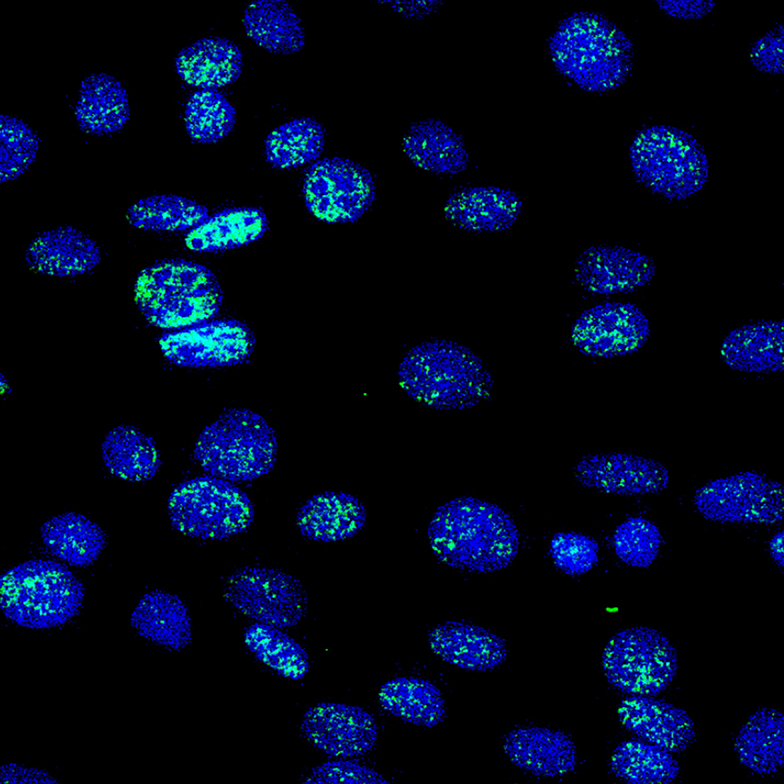

Supplement: Supplementary file 3 [file Data_Sheet_2.zip › Fig.6/Fig.6D/S/NRF-1 S confocal.jpg]

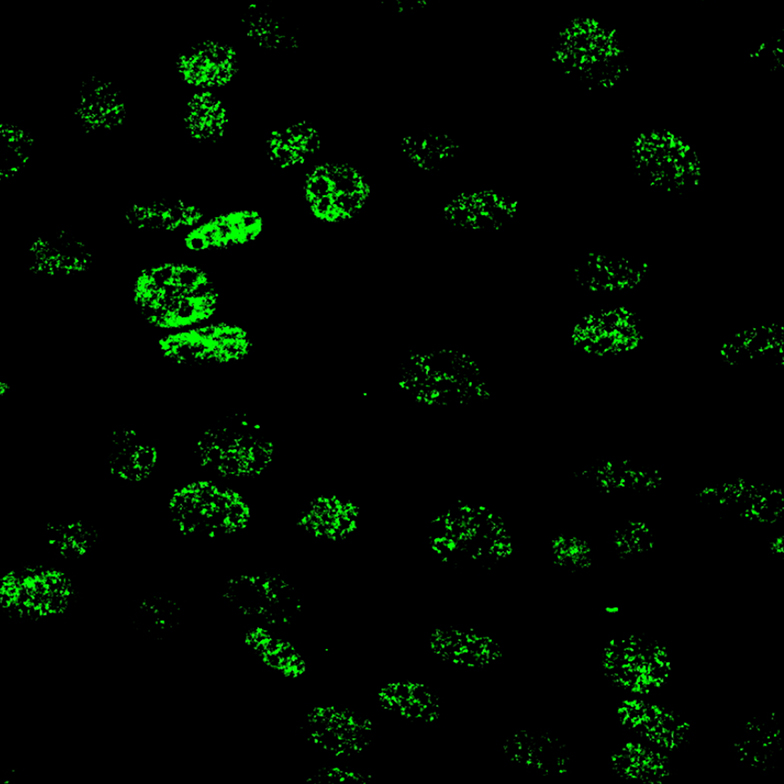

Supplement: Supplementary file 3 [file Data_Sheet_2.zip › Fig.6/Fig.6D/S/NRF-1 S.jpg]

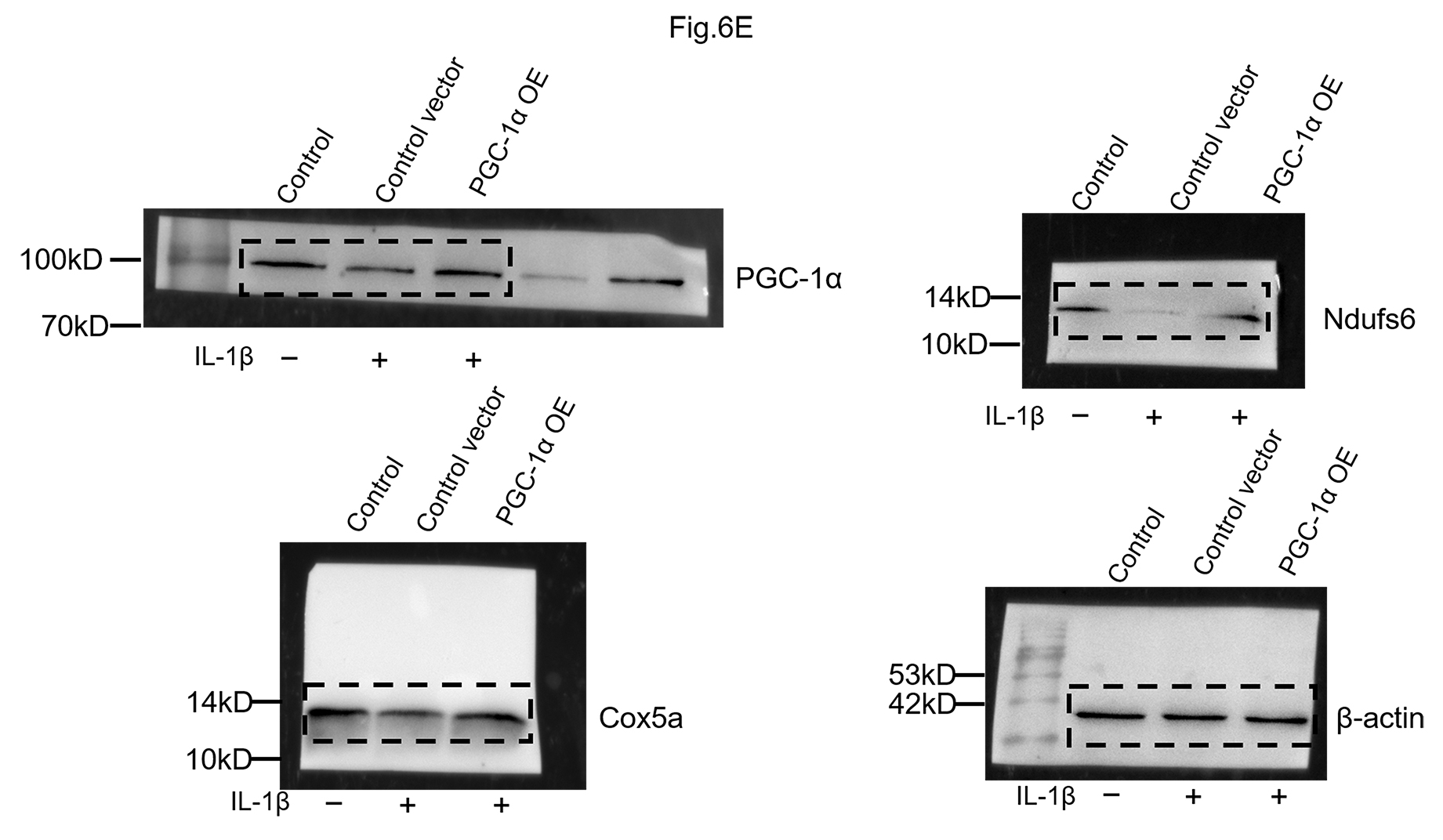

Supplement: Supplementary file 3 [file Data_Sheet_2.zip › Fig.6/Fig.6E/Fig6E.jpg]
