# Supplementary material for: Hippocampus-Based Mitochondrial Respiratory Function Decline Is Responsible for Perioperative Neurocognitive Disorders
Source: Front Aging Neurosci. 2022 Feb 9;14:772066. doi: 10.3389/fnagi.2022.772066 (PMC8865419; doi:10.3389/fnagi.2022.772066)
Supplement: Supplementary file 2 [file Data_Sheet_1.zip › Fig.2/RNA-seq rawdata adress.docx]

<https://www.ncbi.nlm.nih.gov/geo/subs/>

token: mfmnykkatfuljep
